# A persistent prefrontal reference frame across time and task rules

**DOI:** 10.1038/s41467-024-46350-4

**Published:** 2024-03-08

**Authors:** Hannah Muysers, Hung-Ling Chen, Johannes Hahn, Shani Folschweiller, Torfi Sigurdsson, Jonas-Frederic Sauer, Marlene Bartos

**Affiliations:** 1 Institute for Physiology I, Medical Faculty, Albert-Ludwigs-University Freiburg, Freiburg im Breisgau, Germany; 2 Faculty of Biology, Albert-Ludwigs-University Freiburg, Freiburg im Breisgau, Germany; 3 Institute of Neurophysiology, Goethe University Frankfurt, Frankfurt am Main, Germany; 4 Sleep-Wake-Epilepsy Center and Center for Experimental Neurology, Department of Neurology, Inselspital, Bern University Hospital, University of Bern, Bern, Switzerland

**Keywords:** Neural circuits, Cellular neuroscience

## Abstract

Behavior can be remarkably consistent, even over extended time periods, yet whether this is reflected in stable or ‘drifting’ neuronal responses to task features remains controversial. Here, we find a persistently active ensemble of neurons in the medial prefrontal cortex (mPFC) of mice that reliably maintains trajectory-specific tuning over several weeks while performing an olfaction-guided spatial memory task. This task-specific reference frame is stabilized during learning, upon which repeatedly active neurons show little representational drift and maintain their trajectory-specific tuning across long pauses in task exposure and across repeated changes in cue-target location pairings. These data thus suggest a ‘core ensemble’ of prefrontal neurons forming a reference frame of task-relevant space for the performance of consistent behavior over extended periods of time.

## Abstract (web summary)

The prefrontal cortex (PFC) is involved in rule-coding and long-term memory. Here, the authors identified a stably active neuronal ensemble in the mouse medial PFC in an olfaction-guided spatial memory task, which showed stable tuning to task features across time, rule-reversal, and context changes.

## Introduction

The question of how temporally stable behavior is mediated by brain activity remains enigmatic. Two opposing views are supported by experimental findings: One framework emphasizes gradually changing neuronal representations in response to identical stimuli (‘representational drift’). Such representational drift has been observed during perceptual tasks in the sensory cortex^1–3^, and during navigational tasks in the associational cortex^4,5^ and the hippocampal area CA1^6^. The finding of representational drift raises the question how changing neuronal tuning might produce stable behavioral performance. Several solutions to this problem have been proposed, including stable readout from non-randomly drifting population codes^5^, consistent population codes residing in high-dimensional manifolds rather than in the activity of individual neurons^7^, and ‘self-correcting’ assembly codes that reassign new members^8^. A drifting coding regime is, thus, in principle compatible with stable perception and cognitive behavior. As an alternative to drift, a fixed association between sensory inputs and neuronal responses leading to stable activity of a set of neurons encoding the behavioral output has been observed. Such temporally stable neuronal responses to recurrent stimuli have been described in the sensory cortex^9,10^, in the dentate gyrus of the hippocampus^6^, and during recall of fear-related memory traces from neocortical long-term stores^11^ (‘engram’).

The prelimbic region of the mPFC is necessary to support the execution of spatial working memory and decision-making^12–14^. While several studies have investigated multiple prefrontal subregions and their responses during memory and decision-making tasks within single experimental days^12,15–19^, the dynamics of mPFC activity over extended time periods have remained unexplored. It is thus unclear whether prefrontal activities follow a stable or a dynamic, drifting encoding regime on the timescale of weeks. To address this open question, we monitored the activity of prefrontal neurons in the prelimbic and anterior cingulate area over several weeks while mice performed an olfaction-guided two-choice task probing decision-making and short-term memory.

## Results

### Task-related activity remains stable over weeks

**Fig. 1:**
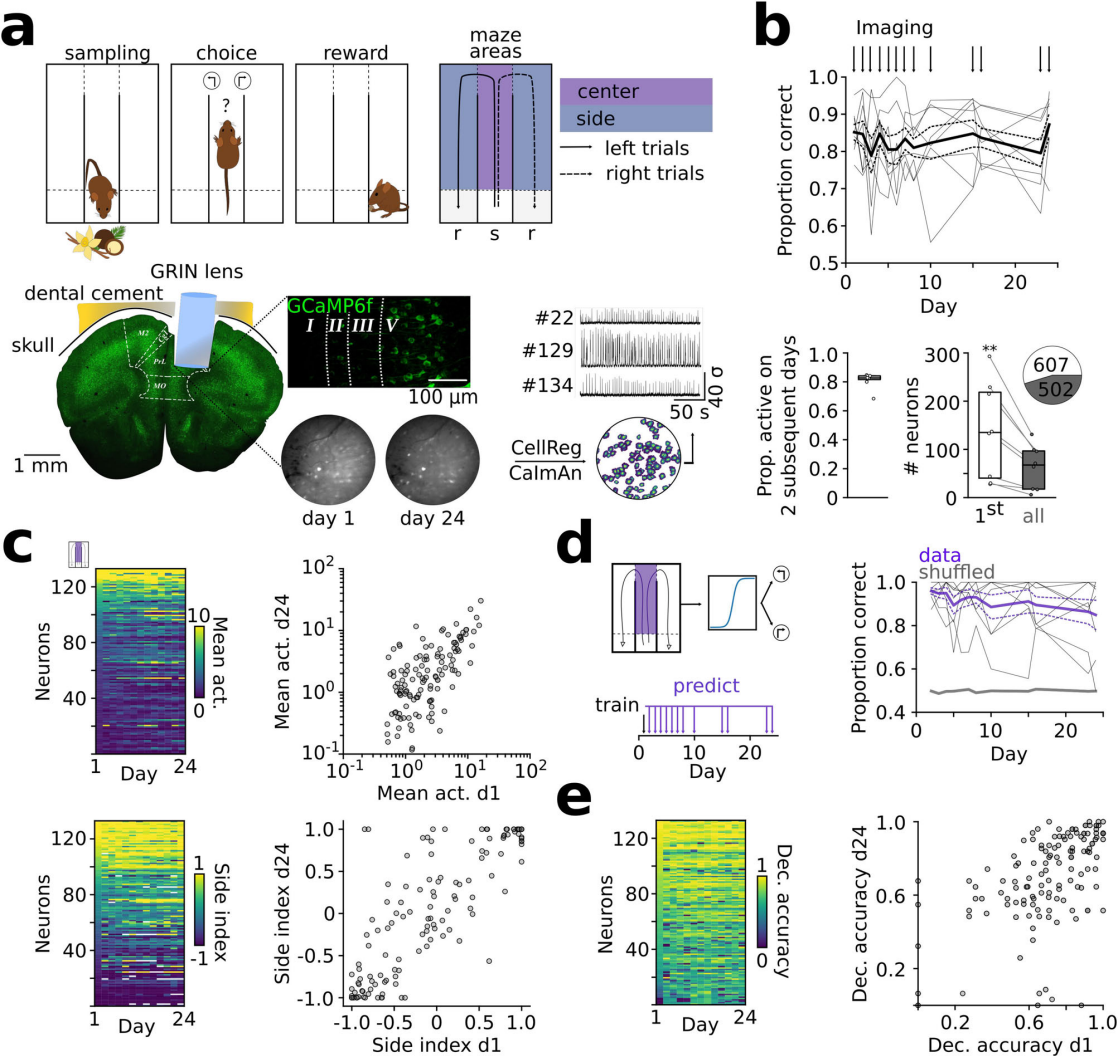
Active prefrontal ensembles stably encode choice over time. **a** Top: schematic of behavioral task. Mice learn to associate two odors (s: sampling) with reward sites at left and right arms (r: reward). Bottom: schematic of the GRIN lens implantation for 1-photon calcium imaging in the mPFC and longitudinal registration of recorded neurons. **b** Top: stable behavioral performance (F = 0.93, *p* = 0.527, repeated measures ANOVA). Arrows indicate imaging days. Bottom: proportion of active neurons during two subsequent days (left) and number of neurons active on the first vs. all imaging days (right, *t* = 3.85, *p* = 0.006, 2-sided paired *t*-test). **c** Top: Stable mean calcium activity in the center arm over days (averaged for left and right, sorted: day 1, Spearman’s r last vs. first: 0.635, *p* = 2*10^−16^). Bottom: Stable preference for activity during left or right trials (side index, Spearman’s r last vs. first: 0.813, *p* = 7*10^−32^, *n* = 133 neurons). 2-sided correlation tests. **d** Decoding of behavioral choice from center arm calcium activity. The models were trained on day 1. Decoding remained significant versus shuffled data (F = 177.17, *p* = 3*10^−6^) with no decoding accuracy-time interaction effect (F = 1.38, *p* = 0.276, 2-way repeated measures ANOVA). Purple: data, gray: shuffled. **e** Within-day decoding accuracy of single neurons remains correlated over days (sorted for day 1, Spearman’s r last vs. first: 0.573, *p* = 6*10^−13^, *n* = 133 neurons). 2-sided correlation tests. Boxes show median and upper/lower quartiles. Circles are individual mice (**b**) or cells (**c**, **e**). Lines indicate mean ± sem, thin lines show individual mice (*n* = 8 for all comparisons). Source data are provided as a Source Data file.

We performed 1-photon calcium imaging in Thy1-GCaMP6f-mice^20^, which express GCaMP6f predominantly in cortical layer 5 pyramidal neurons^20^ (Fig. 1a, Supplementary Fig. 1). This transgenic approach enabled us to reliably image the same set of deep layer neurons across prolonged periods of time without cytotoxicity due to viral expression (Supplementary Movie 1). Retrograde tracing revealed that GCaMP6f was sparsely expressed in both intratelencephalic and pyramidal tract neurons, indicating that both main classes of layer 5 projection neurons were present in the recorded population (Supplementary Fig. 2a, b). In the task, the animals learned to associate two odors (vanilla or coconut) delivered in the central stem of a figure M-maze with reward sites located at the end of the left and right side arm, respectively (Fig. 1a). Lens implantation in the mPFC resulted in moderate levels of gliosis and did not affect learning of the behavioral task (Supplementary Fig. 2c, d). Moreover, electrophysiological recordings performed during the task revealed comparable activity profiles of mPFC neurons, suggesting that lens implantation left the microcircuit properties of the mPFC intact (Supplementary Fig. 2e, f).

Upon learning, the animals displayed stable behavioral performance, which persisted over several weeks (Fig. 1b). Recording from the same set of prefrontal neurons, thus, provides the opportunity to assess the dynamics of population activity during repeated execution of the learned task. Longitudinal registration revealed that 82.9 ± 0.5% of layer 5 neurons could be registered from one day to the next (Fig. 1b, Supplementary Fig. 3). Registration across all days showed that 47.8 ± 2.5% of neurons were detected as active cells on each day throughout the ~3 week recording period (63 ± 16 neurons per mouse, 8 mice, 13 recording days over 24 days in total, Fig. 1b), indicating that a subset of neurons repeatedly activates during task exposure. Across days, repeatedly active neurons retained stable activity levels as quantified from calcium transients during center arm travel (Fig. 1c, upper; see Supplementary Fig. 4 for a quantification of activities in the side arm and for different activity threshold values, which gave similar results). We observed that many prefrontal neurons differed in their activity during left and right trials. This was quantified with a side index ranging from −1 (only active during left trials) to 1 (only active during right trials). The side index of individual neurons remained similar over recording days (Fig. 1c, lower). These results prompted us to test whether behavioral choice can be decoded from the same set of neurons over days. We trained models on calcium activity on the first day and decoded the target locations for each mouse on the following days. The decoder allowed the prediction of trial outcome up to the last tested day with high accuracy, irrespective of the chosen decoding model (Fig. 1d, Supplementary Fig. 5e). Similar high decoding quality was obtained using calcium transients or the rising phase of transients instead of mean calcium signals, or when the decoding analysis was restricted to time points in the central arm before the spatial trajectories deviated for left and right turns, presumably corresponding to the decision point of the animal^21^ (Supplementary Fig. 5h). Moreover, similar decoding results were obtained when the model was trained on the last recording day to predict trial outcome on previous days (Supplementary Fig. 5d). While neurons with large side index were most effective at decoding trial identity, only ~10 randomly selected cells were sufficient to decode trial outcome at ~90% accuracy within a day and ~15 neurons sufficed to decode trial outcome at ~80% accuracy across the full 24 days (Supplementary Fig. 6c, e). In addition, we quantified the decoding of trial outcomes on a day-by-day basis using individual neurons of the repeatedly active ensemble. This analysis revealed that the decoding performance of task-active neurons was highly correlated over time (i.e., a neuron with large decoding accuracy on day 1 also showed large accuracy on day 24, Fig. 1e). There was no correlation between the change in single-cell decoding accuracy over days and the position of peak activity of the neurons in the arena (Supplementary Fig. 6f). These data jointly indicate that the same set of prefrontal layer 5 neurons maintains similar choice-specific information over weeks.

### Trajectory-specific spatial tuning is preserved over time

**Fig. 2:**
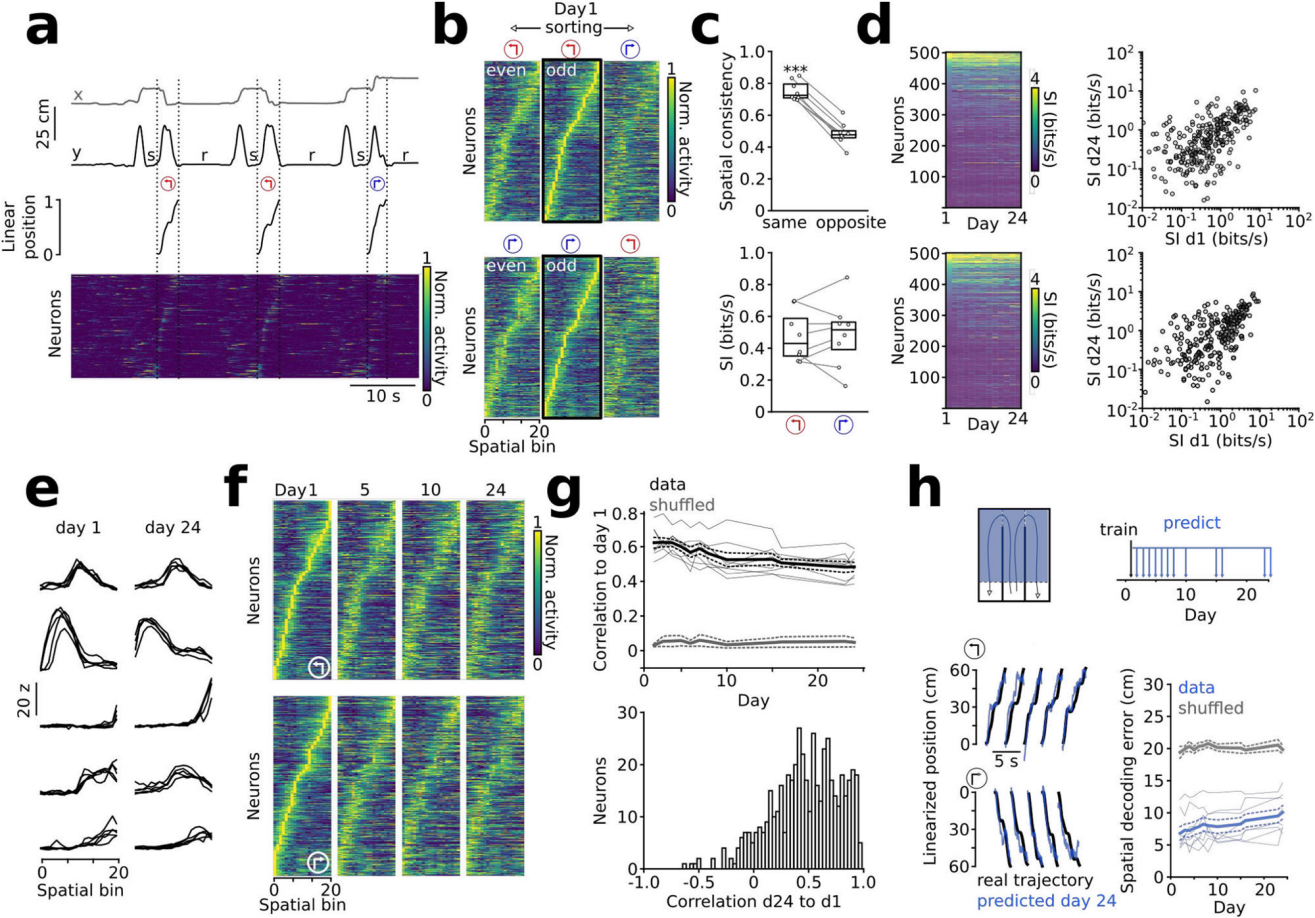
Persistent trajectory-specific tuning over weeks. **a** Example of x/y-coordinates, linearized position, and normalized calcium activity (sorted by location of peak activity during left trajectories). s: sampling; r: reward. **b** Spatial tuning functions during odd and even left and right runs within the first recording day (*n* = 1109 neurons). **c** Top: Spatial correlation of odd vs. even runs (consistency) is higher within trials of the same vs. opposite direction (*t* = 18.21, *p* = 3*10^−7^). Bottom: Spatial information (SI) is comparable during left and right trials (*t* = −0.28, *p* = 0.791). Two-sided paired *t*-tests. **d** Spatial information (SI) remains consistent across days during left (top, *r* = 0.654, *p* = 10^−62^) and right runs (bottom, *r* = 0.648, *p* = 10^−61^, Spearman’s correlation coefficients day 24 vs. day 1, *n* = 502 neurons). 2-sided correlation tests. **e** Examples of spatially binned activities during five superimposed individual left runs on day 1 and day 24. **f** Spatial tuning functions sorted for the peak location on day 1 (for left and right trajectories, respectively) show large spatial stability across days. **g** Top: Average trajectory-specific spatial correlation to day 1. Correlations decayed over time (F = 16.48, *p* = 10^−6^ for the correlation-time interaction) but remained significant vs. shuffled data to the last day (*t* = 11.29 to 18.42, *p* = 10^−5^ to 3*10^−7^, 2-way repeated measures ANOVA followed by paired *t*-tests with Šidák correction). Bottom: Distribution of correlation coefficients of individual cells. **h** Decoding of the animals’ linearized position. Left: Example of the prediction on day 24 using a model trained on day 1 (blue). Right: Predictions decayed over time (F = 3.48, *p* = 6*10^−4^ for the decoding error-time interaction) but remained significant vs. shuffled data (gray, *t* = −7.32 to −13.00, *p* = 2*10^−4^ to 6*10^−6^, 2-way repeated measures ANOVA followed by paired t-tests with Šidák correction). Boxes show median and upper/lower quartiles. Circles are individual mice (**c**) or cells (**d**). Lines in (**g**) and (**h**) show mean ± sem, thin lines are individual mice. Left and right trajectories were correlated separately and pooled subsequently (*n* = 8 for all comparisons). Source data are provided as a Source Data file.

Prefrontal neurons show spatially tuned firing during cognitive tasks^14,16–18,22^. Whether spatial tuning remains consistent over weeks of task execution, however, has not yet been assessed. In light of the stable side indices of prefrontal neurons across days, we thus asked whether a consistent trajectory-specific spatial map might underlie trial-specific activity patterns. In agreement with previous reports^14,16–18,22^, the spatially binned activity of task-active layer 5 neurons tiled the entire extent of the arena (Fig. 2a, b, for electrophysiological control data see Supplementary Fig. 2g). Consistent with the side preference of some neurons, spatial tuning functions were more strongly correlated between trajectories towards the same as compared to the opposite side (Fig. 2b, c). We therefore determined trajectory-specific tuning functions of cells for left and right trajectories and compared them over multiple days. Spatial information content remained correlated over recording days (Fig. 2d), suggesting that neurons retain their individual spatial tuning strength over weeks. Moreover, daily active neurons maintained significantly correlated trajectory-specific tuning functions throughout days 1–24 (Fig. 2e, f, see Supplementary Fig. 7 for responses during individual runs). Correlation to the first day decayed at a rate of ~0.006/day, consistent with a slow representational drift (Fig. 2g). Compared to surrogate data, in which representational drift was simulated by a cumulative shift of spatial tuning functions over days, strong correlations to day 1 persisted over a large parameter space (Supplementary Fig. 7b). Additional analysis of population vectors and the geometrical structure of the population activity^3^ confirmed that decorrelation among the responses of individual neurons emerged only slowly over time (Supplementary Fig. 7d, e). Despite the observed mild drift, trajectory-specific spatial correlation to the first day remained, on average, at ~0.5 on day 24, markedly higher than shuffled control data (Fig. 2g, Supplementary Fig. 7b, c). We therefore tested whether the position of the animal on the track can be predicted from calcium activity of the first day. Using models trained on day 1, the animals’ position during outward travel could be reliably decoded up to the last tested day (Fig. 2h, see Supplementary Fig. 8 for analyses using transients or rising phases as activity measures, for different decoding models, and for decoding based on models trained on the last instead of the first recording day). Neurons with high spatial information contributed most to decoding accuracy (Supplementary Fig. 8b). Thus, our data shows a core ensemble of task-related layer 5 neurons with stable trajectory-specific tuning over weeks. Taken together, our data point to a temporally stable representation of task space.

### Representation of task space is most strongly influenced by linearized positions

To identify how different behavioral parameters influence the representation of task space, we used a generalized linear model^14^ (GLM) to disentangle the contribution of (linearized) position, speed, and goal location to calcium activity over the entire trial duration.

**Fig. 3:**
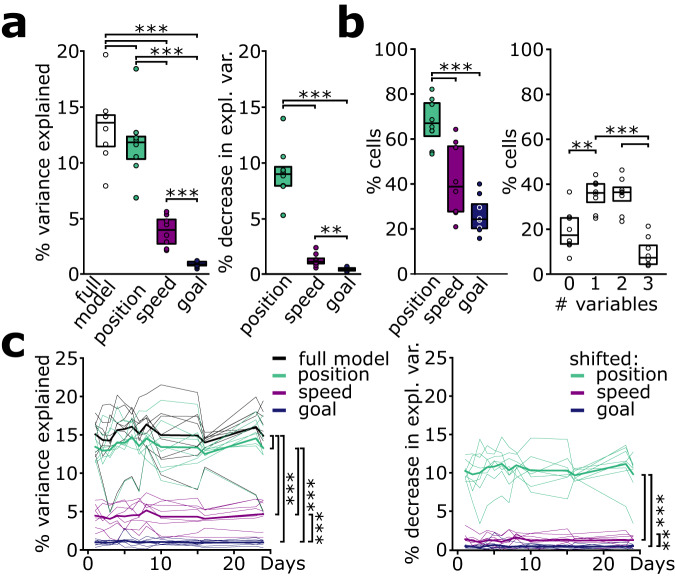
Neuronal activity is most strongly influenced by spatial position. **a** Position is the best predictor for calcium activity. Left: Explained variance of GLMs using all or single predictors (F = 93.71, *p* = 3*10^−12^; full (white) vs position (green): *t* = 8.19, *p* = 8*10^−5^, full vs speed (purple): *t* = 11.23, *p* = 10^−5^, full vs goal (blue): *t* = 10.05, *p* = 2*10^−5^, position vs speed: *t* = −9.42, *p* = 3*10^−5^, position vs goal: *t* = 9.16, *p* = 4*10^−5^, speed vs goal: *t* = 5.82, *p* = 6*10^−4^). Right: Decrease in explained variance with a single predictor shifted in time (F = 87.99, *p* = 10^−8^; position vs speed: *t* = −9.39, *p* = 3*10^−5^, position vs goal: *t* = 9.62 *p* = 3*10^−5^, speed vs goal: *t* = 3.67, *p* = 0.008). **b** Most cells encode for position (green; speed: purple; goal: blue) and for one or two variables in total. Left: Percentage of cells significantly modulated by the different predictors (F = 38.34, *p* = 2*10^−6^; position vs speed: *t* = −7.08, *p* = 2*10^−4^, position vs goal: *t* = 8.70, *p* = 5*10^−5^, speed vs goal: *t* = 2.71, *p* = 0.030). Right: Number of variables they are modulated by (F = 16.35, *p* = 10^−5^; 0 vs 1: *t* = −4.80, *p* = 0.002, 0 vs 2: *t* = −2.74, *p* = 0.029, 0 vs 3: *t* = 1.84, *p* = 0.108, 1 vs 2: *t* = −0.02, *p* = 0.984, 1 vs 3: *t* = 5.83, *p* = 6*10^−4^, 2 vs 3: *t* = 16.67, *p* = 7*10^−7^). **c** The contribution of predictors is stable across days. There is a main effect of model type (full model: black; position: green, speed: purple; goal: blue), with position outperforming the other single predictor models (left, F = 210.13, *p* = 8*10^−16^; full vs position: *t* = 6.62, *p* = 3*10^−4^, full vs speed: *t* = 15.93, *p* = 9*10^−7^, full vs goal: *t* = 14.64, *p* = 2*10^−6^, position vs speed: *t* = −15.32, *p* = 10^−6^, position vs goal: *t* = 15.43, *p* = 1*10^−6^, speed vs goal: *t* = 6.78, *p* = 3*10^−4^) and the largest decrease in explained variance for the position-shifted model (right, F = 245.46, *p* = 10^−11^; position vs speed: *t* = −12.29, *p* = 10^−6^, position vs goal: *t* = 17.28, *p* = 5*10^−7^, speed vs goal: *t* = 3.32, *p* = 0.013). In both analyses, ANOVA identified no effect of time (F = 1.49, *p* = 0.146; F = 1.12, *p* = 0.358) nor model*time interaction (F = 1.13, *p* = 0.287; F = 0.96, *p* = 0.526), suggesting stable contribution of the individual predictors over time. Thick lines indicate mean across animals, thin lines are individual mice. One-way repeated measures ANOVA (**a**) and (**b**), two-way repeated measures ANOVA (**c**), followed by paired *t*-tests with Šidák correction. Boxplots show median and lower/upper quartile of data, circles show individual mice (*n* = 8 for all comparisons). Source data are provided as a Source Data file.

On the first imaging day, the full model containing all three predictors explained 13.2 ± 1.2% of the variance of the calcium signal (Fig. 3a). To identify the contribution of the individual variables, we fit reduced models composed of only a single predictor to the data. This analysis revealed that position accounted for most of the variance, with lower contributions from speed and goal location (Fig. 3a, left). To corroborate this finding, we performed the inverse analysis by randomly time-shifting one predictor from the full model and quantified the reduction in explained variance in those reduced models. Consistently, models with shifted position showed the largest decrease in explained variance, followed by speed and goal location (Fig. 3a, right). Moreover, 67.6 ± 3.8% of the cells were significantly influenced by the predictor position, whereas only 41.6 ± 5.8% of the neurons were significantly influenced by speed and 26.2 ± 2.9 by goal location (Fig. 3b, left). In total, 19.5 ± 3.3% of cells were not significantly influenced by any predictor and 9.7 ± 2.3% by all three parameters. Most of the cells were either influenced by one or two of the predictors with 35.3 ± 2.4% and 35.4 ± 2.7%, respectively (Fig. 3b, right). Thus, linearized position carries the most explanatory power for neuronal activity and most cells encode for one or two of the investigated variables.

We next asked whether the dominance of position in terms of explanatory power for a cell’s calcium signal is preserved across days. Full models, single predictor models or models with one behavioral variable shifted in time showed a similar percentage of explained variance across days in our ensemble of repeatedly active neurons (Fig. 3c), suggesting stable encoding of the behavioral variables across time in the imaged cell population. The stable representation of task space in principal cells, thus, represents a combination of all investigated variables including position, speed, and goal location, with the largest contribution of linearized position.

### Stable representation of task space emerges during learning

**Fig. 4:**
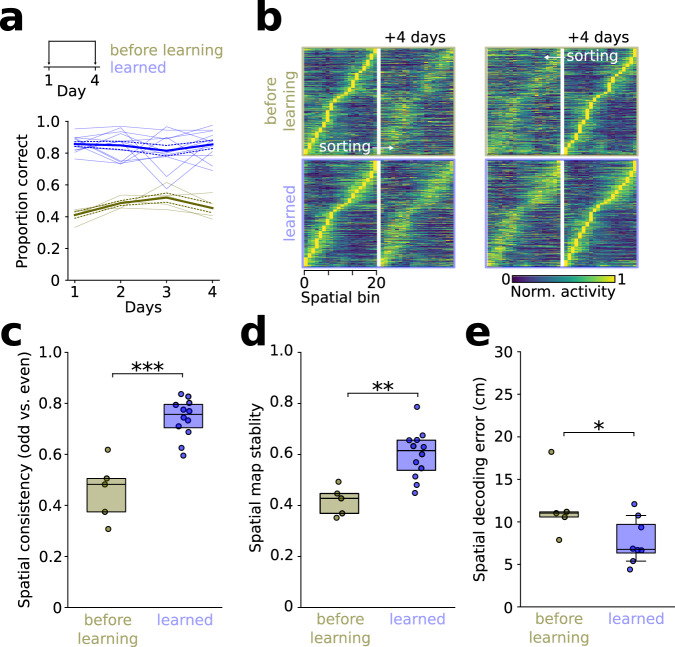
Stable reference frames emerge during learning. **a** Top: Trajectory-specific map stability was compared before and after learning both within the first day (consistency) and over 2 recording sessions 4 days apart (stability). Bottom: behavioral performance during before learning (gold) and learned blocks (blue). **b** Trajectory-specific tuning functions on day 1 and 4 during before learning (top: *n* = 923 neurons) and learned conditions (bottom: *n* = 1611 neurons). **c** Learning increases spatial consistency. *T* = −5.93, *p* = 3*10^−5^, 2-sided unpaired *t*-test, *n* = 5 and 12 mice. **d** Reduced spatial stability in the before learning condition (*t* = −3.98, *p* = 0.001, *n* = 5 and 12 mice, 2-sided unpaired *t*-test). **e** Larger spatial decoding error across 4 days in the before learning compared to the learned condition (*t* = 2.23, *p* = 0.047, *n* = 5 and 8 mice with >50 neurons, 2-sided unpaired *t*-test). Boxes show median and upper/lower quartiles. Circles represent individual mice. Source data are provided as a Source Data file.

To test whether the emergence of a stable representation of task space requires learning of the task rules, we imaged prefrontal neurons in a separate cohort of mice during initial task exposure before the animals had learned the rule (before learning group, average task performance: 47 ± 2%, as opposed to learned with ≥70% correct, Fig. 4a, b). This stage in the learning process was chosen because the animals traversed the maze in consistent outward trajectories, allowing us to construct directional spatial tuning functions and to compare their temporal stability with the learned state (Supplementary Fig. 9). Average spatial information content and the proportion of repeatedly active neurons was comparable between both conditions (Supplementary Fig. 9a, b). However, when data from the first day of each condition were considered, the consistency of trajectory-specific maps between odd and even runs was significantly lower in the before learning compared to the learned group (Fig. 4c). While trajectories of individual runs were more variable before learning, larger spatial correlation in the learned group persisted when we compared tuning functions for pairs of individual trials with comparable difference in trial duration (Supplementary Fig. 9c, e, g). Moreover, correlation of the directional spatial tuning across four days was lower in the before learning compared to the learned group (Fig. 4d). In line with this observation, spatial errors of decoders trained on the first day and tested on data from the fourth day were significantly larger in the before learning group (Fig. 4e). These data jointly suggest that the prefrontal representation of task space stabilizes during task learning.

### Time rather than experience determines drift in representation of task space

**Fig. 5:**
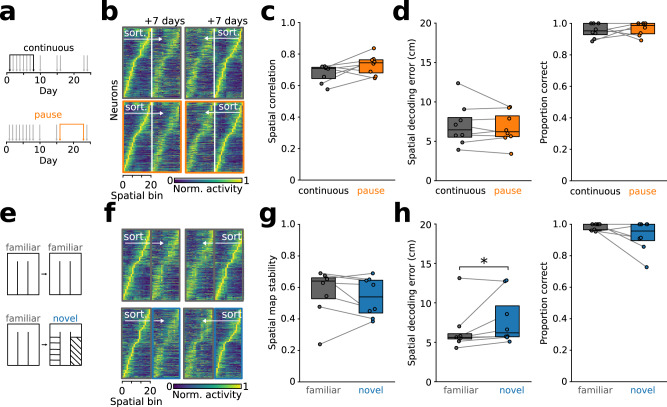
Preserved trajectory-specific tuning across pauses in task execution and across contexts. **a** Schematic of continuous (i.e., daily execution of the task) and pause blocks (i.e., no exposure to the task apparatus over 7 days). Gray arrows show the days on which the mice experienced the task. Black arrows show the compared days for continuous (gray) and pause conditions (orange), separated by 7 days each. **b** Trajectory-specific tuning functions sorted by the first (left) and last day (right) of both continuous and pause pairs. 958 and 945 neurons, *n* = 8 mice. **c** Task pause does not affect trajectory-specific map stability over 7 days (*t* = −2.01, *p* = 0.084, 2-sided paired *t*-test). **d** Similar linearized position decoding (left, training: day 1, testing: day 7, *t* = 0.91, *p* = 0.395, 2-sided paired *t*-test) and prediction of trial outcome based on calcium signals in the center arm (*T* = 8, *p* = 0.600, 2-sided Wilcoxon signed-rank test) across continuous and pause blocks. **e** Schematic of familiar-familiar and familiar-novel pairs of recording days. Trajectory-specific tuning of all cells that were active on both days of the pair were analyzed. **f** Trajectory-specific tuning functions during familiar-familiar and familiar-novel sequence sorted by the first (top) or second day (bottom). 1261 and 1267 neurons. **g** Similar trajectory-specific spatial correlation (*t* = 0.94, *p* = 0.379, 2-sided paired *t*-test) for familiar-familiar (gray) and familiar-novel conditions (blue). **h** Accuracy of position decoding (*T* = 3, *p* = 0.039) and prediction of trial outcome based on calcium signals in the center arm (*T* = 2, *p* = 0.138, 2-sided Wilcoxon signed-rank tests) for familiar-familiar and familiar-novel conditions. Boxes show median and upper/lower quartiles. Circles are individual mice (*n* = 8 mice for all comparisons). Source data are provided as a Source Data file.

We next investigated whether the small changes in trajectory-specific tuning observed after learning depend on repeated exposure to the task (i.e., experience) or on the progression of time. In task-proficient mice, we compared the first and last recording of a sequence of 7 sessions of daily task exposure (continuous) with pairs of recording days separated by a 1-week pause in task execution (pause, Fig. 5a). During the pause, mice were kept in the holding facility without exposure to the experimental room or the task arena. There was no difference in the correlation of trajectory-specific tuning in continuous compared to pause pairs (Fig. 5b, c). Consistently, models trained on the first day of each pair could predict both position and trial outcome on the second day equally well in continuous and pause blocks (Fig. 5d). These results suggest that elapsed time between sessions rather than experience underlies the observed slow representational drift in the mPFC.

### Representation of task space generalizes across contexts

To test whether the representation of task space differentiates between task arenas, we assessed the stability of trajectory-specific tuning of prefrontal cells in task-proficient mice exposed to a visually modified (novel) arena (Fig. 5e). The novel arena differed from the familiar one in vertical and horizontal stripes and dots outlining the arena. Trajectory-specific tuning stability was comparable between familiar-familiar and familiar-novel pairs of subsequent recording days (Fig. 5f, g). Concurrently, models trained on data obtained in the familiar arena could predict the animals’ spatial position from calcium activity of the same set of neurons in the novel arena, albeit with mildly larger spatial decoding error (Fig. 5h, left). Finally, trial outcome could be predicted equally well in both familiar and novel arenas based on decoders trained in the familiar context (Fig. 5h, right). These results suggest that the representation of task space generalizes across contexts.

### Representation of task space remains consistent across changes in the reward rule

**Fig. 6:**
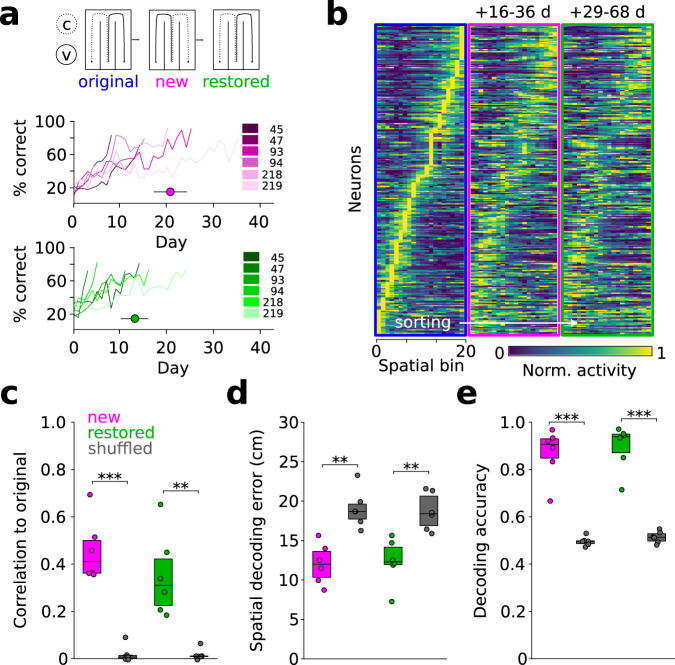
Trajectory-specific tuning persists across rule changes. **a** Top: Schematic of original, new, and restored rule conditions. Bottom: Corresponding learning performance color-coded for individual mice (*n* = 6 mice, purple: new rule, green: restored rule). Circles indicate average duration ± sem to criterion. C: coconut, V: vanilla. **b** Trajectory-specific tuning functions of all cells during original, new, and restored rules sorted by the original rule (*n* = 272, only left trajectories are shown). Since the last recording day in the original rule, 16–36 and 29–68 days passed until the mice had learned the new and restored rule, respectively. **c** Trajectory-specific correlation to the original rule decays over time but remains significant vs. shuffled (gray) during both new (*t* = 10.37, *p* = 10^−4^) and restored rules (*t* = 5.17, *p* = 0.003, time-correlation interaction effect: F = 13.68, *p* = 0.014, two-way repeated measures ANOVA followed by paired *t*-tests with Šidák correction). **d** Models trained on data obtained during the original rule allow the prediction of the animals’ linearized position during new (versus shuffled: *t* = −5.30, *p* = 0.003) and restored rules (*t* = −5.40, *p* = 0.003, two-way repeated measures ANOVA followed by paired t-tests with Šidák correction). **e** Models trained on the original rule allow the decoding of behavioral choice during new (versus shuffled: *t* = 7.78, *p* = 6*10^−4^) and restored conditions (*t* = 7.76, *p* = 6*10^−4^, two-way repeated measures ANOVA followed by paired *t*-tests with Šidák correction). For all comparisons, left and right trajectories were correlated separately and pooled subsequently. Boxes show median and upper/lower quartiles. Circles represent individual mice (*n* = 6 for all comparisons). Source data are provided as a Source Data file.

Since our data suggest substantial stability of neuronal responses upon learning, we asked how newly learned cue-reward associations would be incorporated into that stable coding regime. We inverted the association of left and right reward sites with the two odors in a subset of mice (new rule), and, upon learning of the new rule, trained the same mice back again on the initial reward rule (restored rule, 6 mice, Fig. 6a). Despite an observable drift, trajectory-specific tuning representing the learned new rule retained high similarity to the original state, with considerable correlation to the original tuning pattern surviving until the mice had learned the restored rule (up to 68 days after learning of the original rule; Fig. 6b, c, Supplementary Fig. 10b). In line with this data, position during left and right trajectories could be decoded during new and restored states using a model trained on the original rule data (Fig. 6d). Moreover, the neurons’ side index remained correlated to the original rule during both new and restored conditions (Supplementary Fig. 10c, d). Consistently, a model trained on data upon learning of the original rule allowed the decoding of trial outcome during outward travel in both new and restored states (Fig. 6e). These data jointly indicate that the tuning of mPFC neurons persists across alterations in the cue-outcome contingency, reminiscent of a slowly drifting representation of task space.

## Discussion

Previous work showed consistent spatial tuning of prefrontal neurons during spontaneous behavior within one experimental session^23^, during subsequent trials of tasks under the same rule^15,24,25^, and during spatial reversal learning^24^. Here, we extend upon these observations by reporting a stable representational structure of prefrontal tuning to the trajectory in the maze (i.e., space) up to several weeks (see Supplementary Fig. 11 for a summary of the findings). Our data imply that prefrontal neurons show only weak representational drift, even under conditions of spatial reward rule inversions. Decoding analyses moreover suggest that downstream reader networks might infer behavioral choice from the activity of a few prefrontal neurons that are part of a temporally stable core ensemble, which we identified here. The core ensemble might provide an efficient way to drive learned, stereotyped motor responses in a task, and might thus contribute to consistent skilled behavior over weeks. Our results are reminiscent of stable responses in orbitofrontal cortex during the execution of a non-spatial go/no-go task^26^ but stay in contrast to recordings from the posterior parietal cortex, another associational neocortical area, where prominent representational drift has been observed in a spatial short-term memory task^4^.

In line with a recent theoretical publication suggesting that reference frames might form the basis of location-feature mapping underlying learning and memory formation in neocortical networks^27^, we propose that mPFC neurons generate a reference frame of task-relevant space, which, once formed during learning, provides a stable scaffold of task space over the course of weeks. In this task space, trajectory-specific positional coding seems to be abundantly and stably encoded. It remains to be investigated in future work whether the stable reference frame generalizes to non-spatial tasks and extra-dimensional rule shifts.

Although 1-photon imaging in transgenic Thy1-GCaMP6f mice is a reliable method to investigate cell populations over extended time periods in freely moving animals^27^, it has its limitations. First, the implanted lens causes a lesion to the neocortex and we cannot unequivocally exclude that our results might be influenced by altered neuronal connectivity or local network changes caused by lens implantations. We observed no behavioral differences in task performance or learning between controls and upon lens implantation (Supplementary Fig. 2d). It remains, however, to be tested whether learning and/or execution of the behavioral task in this study depends on the intact mPFC circuitry in unimplanted animals. Second, the observation that some cells have been found to be active on one day but not on another day might be a reflection of a true biological phenomenon (i.e., neurons turning inactive between sessions), a technical confound (e.g., neurons moving out of focus), or a mixture of both. To circumvent this limitation, we analyzed repeatedly active cells, which were found across all imaging days. Although we could image the same area of interest with high reliability, we cannot fully exclude the possibility that some cells, which could not be registered across days, might have altered their trajectory-specific tuning. Second, GCaMP6f signals in the transgenic mouse line used in this study were largely restricted to deep mPFC layers (Supplementary Fig. 1). Previous work indicated comparable spatial tuning characteristics of superficial and deep layer mPFC cells during navigation in virtual^23^ or real worlds in individual sessions^17,18^. Future work will be needed to test whether superficial layer neurons show similar stable tuning responses across weeks.

Upon learning the task, we observed a stabilization of trajectory-specific tuning. We hypothesize that this is based on a stabilization of the reference frame relevant for the animal to navigate in the task. Learning results in more stereotypical behavioral trajectories, and behavioral variability has been linked with variability in the neuronal representation^28^. However, we did not observe a correlation of trial duration with trajectory-specific tuning correlations (neither within day nor across days). Moreover, larger trajectory-specific tuning correlations in the learned group persisted when we restricted the analysis to pairs of trials with low variability in inter-trial duration (Supplementary Fig. 9g). These results thus argue against the stabilization observed across learning being merely caused by more consistent behavior over time. Still, an alternative hypothesis that warrants direct testing in future is that prolonged experience of the arena per se might cause a stabilization of the reference frame even in the absence of learning a specific task.

Once learned, trajectory-specific tuning remained consistent across pauses in task execution, suggesting that the progression of time per se, rather than experience of the task, might underlie the slow representational drift in the mPFC. This finding stays in marked contrast to observations in the hippocampus, characterized by remapping of place fields both across trials on one day and across days within the same arena^29,30^. In line with the notion of time rather than experience as the dominating factor underlying representational drift in the mPFC, changes in trajectory-specific tuning were not substantially accelerated by rule reversal learning and generalized to a novel, visually distinct arena. Indeed, generalization among contexts is supported by previous work further emphasizing abstract representations of task context in the mPFC^16,31,32^.

Whether the observed stability of trajectory-specific tuning is internally generated in the mPFC or inherited from another brain region remains to be determined. It is plausible that a sequence of activated prefrontal neurons along task trajectories is acquired during learning by recurrent weight changes within the mPFC network. These synaptic weights might be relatively robust against further updating, such as during daily task exposure or post-learning rule changes in our goal-oriented olfactory learning task. Similarly robust encoding of information has been previously observed in the form of fear memory-related prefrontal engrams that reliably reactivate upon reexposure to the fearful context even after weeks of initial experience^11^.

The hippocampal area CA1 is the major source providing spatial information to the mPFC^33,34^. The majority of CA1 neurons change their spatial representation over time^6,35^, suggesting that a transformation of dynamic-to-stable neuronal responses might occur during this passage of spatial information to the mPFC network. The cellular and network processes which might underlie the transmission of spatial information remain, however, to be investigated. Alternatively, a subset of CA1 place cells with temporally consistent place field locations^36^, or spatially tuned neurons in other neocortical areas such as somatosensory^37^ or orbitofrontal cortex^38^ might support stable spatial tuning in mPFC circuits.

Whether the observed stable prefrontal reference frame is necessary for task execution needs to be tested in the future (e.g., by targeting specific cells within this stable framework^39^). The task representation is probably widely distributed across the brain, as has recently been shown for other tasks^40–42^. A resulting important question will be to disentangle to what extent variations in the representational stability across cortical areas might play a role in long-term memory and behavior.

## Methods

### Animals

Thy1-GCamp6f mice^20^ (3 female, 10 male mice) (Jackson Labs #025393) maintained on a heterozygous background by crossing with C57Bl6/J (Jackson Labs #000664) mice were used for the imaging experiments in this study (age at the beginning of the experiments: 12-16 weeks). For retrograde tracing, 9 Thy1-CGamp6f mice were used (2 female, 7 male mice). Additionally, electrophysiological data from 4 male Ai32(RCL-ChR2(H134R)/EYFP) mice (Jackson Labs #012569) used in a previous study^43^ is included. The animals were maintained on a 12 h light-dark cycle with free access to food and water until the start of behavioral training. Mice were food-restricted for behavioral training to 85–90% of their freely feeding body weight. All experiments were performed in agreement with national legislation (licenses G18-145 and G19-145 approved by the Regierungspräsidium Freiburg).

### Behavioral task

Animals were first habituated to the experimental room and the experimenter for at least 3 days by leaving the animals undisturbed in the experimental room for 30 min. Food restriction was started and the animals were introduced to the behavioral arena. Olfactory cues (vanilla: *Dr. Oetker* Vanilla Essence 2 mL, 1:10 diluted in water; coconut: 100% pure coconut oil *Vita D’Or*, liquefied in a warm water bath) were presented at a sniffing port in the central stem of the arena (M-shaped maze, arm length 40 cm). In the first training phase mice learned to nose-poke into the sniffing port, were one of the odors was randomly presented, and to run to the reward location at the end of the side arm to collect a food pellet (20 mg, Dustless Precision Pellets® Rodent, *Bio-Serv* F0071). In this phase only one arm was accessible during each trial. Once the animals learned to sample the odor, to collect the reward and to initiate the next trial by nose-poking, they were trained to make the correct choice to receive a reward. In this second phase both arms were freely accessible. A reward was only given when the correct target arm was chosen. Containers with a mixed coconut/vanilla mixture were placed around the arena to diffuse the odor presented at the sniff port. A camera above the behavioral arena recorded the movement of the animals.

Experiments were performed on two cohorts of mice: In cohort 1, imaging was started when the mice had reached >70% correct for three consecutive days (8 mice). In cohort 2, calcium signals were acquired during task learning (47 ± 2% correct, average over 4 days during rule learning, *n* = 5), and after reaching the criterion (*n* = 4, one mouse of this cohort did not learn the task). The training to criterion took 11–36 days (17 ± 3 days, *n* = 10 mice; for the remaining 2 mice the training was paused after 6 days and resumed 33 days later. These animals reached criterion 24 days after start of retraining). Before lens implantation (see below), the animals were provided with food ad libitum for at least 3 days. 4 weeks after recovery from implantation, the animals were food-restricted and re-trained in the task to criterion (~7 weeks after lens implantation). The effect of rule changes was tested in a subset of mice of cohort 1 (*n* = 6) by inverting the association of the two odors with the spatial target (new rule, 42–50 days after start of the imaging experiments, mean: 49 ± 1). Upon learning of the new rule, the mice were put back on the originally learned rule (restored, 66–93 days after start of the imaging experiments, mean: 79 ± 4). After learning the restored rule (cohort 1, 4 mice) and after learning (cohort 2, 4 mice) 8 mice were additionally exposed to a novel arena, which had the same dimensions as the original arena but distinct visual cues (horizontal and vertical colored stripes and dots along the walls of the side arms). The animals of cohort 2 received lens implants before the start of behavioral training.

### Surgical procedures

For optical access a gradient reflective index (GRIN) lens of 0.5 mm (*n* = 2) or 1 mm (*n* = 11) diameter was used (length: 4 mm, ProView or ProView Integrated, Inscopix). Mice were anesthetized with isoflurane (induction: 3%, maintenance: 1–2% in O_2_) and placed on a heating pad in a stereotaxic frame. After opening the skin, two small holes were drilled in the left and right parietal bone, respectively, and two stabilizing screws (DIN84 A2 M1 × 2) were inserted. Thereafter, a craniotomy with a diameter of ~1 mm was performed over the mPFC (coordinates: 1.7 mm anterior and 0.6 mm lateral of bregma). The animal’s head was tilted by 5° laterally and two orthogonal cuts were applied to the neocortex with a 21 G injection needle to support the penetration of the lens. The lens was slowly lowered to the mPFC (depth: 1.2–1.6 mm from brain surface). The craniotomy was sealed off with Vaseline, and the lens and screws were cemented to the skull using Superbond c&b (Sun Medical). Buprenorphine (BP, 0.1 mg/kg body weight) and Carprofen (CP, 0.1 mg/kg body weight) were injected s.c. prior to and for 2 days after surgery (2–3 injections of BP and 1 injection of CP/day). BP was supplied for 2 days in the drinking water overnight (10 mg/l). For tracing experiments (see below), BP/CP injection was performed prior to surgery followed by a single injection of BP 4–6 h after surgery.

### Retrograde tracing

Red retroBeads (Lumafluor Inc.) were diluted 1:1 in sterile phosphate-buffered saline (PBS). Injection needles were made from glass tubing using a microfilament puller (Flaming Brown). Under general anesthesia (see above), 200 nl of retroBeads were injected at the following coordinates (in mm): (a) mPFC 1.9 anterior, 0.45 lateral of bregma at a depth of 1.7 from brain surface; (b) striatum: 0.5 posterior, 1.5 lateral of bregma at a depth of 1.8 from brain surface; (c) periaqueductal gray/superior colliculus: 4.5 posterior, 0.5 lateral of bregma at a depth of 2.5 from bregma. Mice were perfused 1 week after injection for histological processing. For retrograde tracing analysis, colocalization was visually determined in confocal image stacks using the *cell counter* plug-in of ImageJ (V1.52r).

### 1-photon calcium imaging

1-photon calcium imaging was performed with nVoke and nVista microscopes (Inscopix) at a frame rate of 20 Hz (exposure time: 49.90 ms, gain: 2–5 (mean: 2.6 ± 0.2), LED power: 0.3–1.1 mW/mm^2^ (mean: 0.7 ± 0.1 mW/mm^2^)). The imaging plane was set to a depth to allow imaging of the highest number of cells. In each imaging session mice performed ~40 trials over the course of 15 min. Landmarks such as blood vessels and the location of active cells were used to maintain a consistent field of view (FOV) over days. Calcium imaging and behavioral recording were synchronized either by triggering the microscope by behavioral video acquisition (EthoVision XT 11.5) or by triggering a blue LED in the FOV of the behavioral camera for offline alignment with the calcium data.

### Electrophysiological recording and spike sorting

To compare the calcium signals measured with 1-photon recording to ground-truth data in the absence of lens-induced neocortical lesions, we analyzed previously acquired electrophysiological recordings^43^ from 4 Ai32(RCL-ChR2(H134R)/EYFP) mice performing the same behavioral task. In brief, custom-made tetrodes (California Fine Wire Company, Tungsten 99.95% CS) mounted on a microdrive were implanted at AP 1.9, ML 0.5, DV −1.0/−1.7. Two grounding screws were implanted in the skull ~2 mm around the lambdoid suture, and an additional stabilizing screw in the rostro-medial part of the parietal bone. The mice carried an additional chronic electrode in the olfactory epithelium, which was not analyzed in the context of this study. Broad-band electrophysiological data were acquired with a tethered amplifier (Intan Technologies, RHD2132) using GUI software (Open Ephys) at a sampling frequency of 30 kHz. A common average computed as the mean of 8–10 randomly selected channels was subtracted from all channels. Recording sections containing artifacts were manually identified and removed. Single units were clustered from 0.3–6 kHz bandpass-filtered data using MountainSort^44^. The obtained clusters were first automatically curated based on isolation (threshold of 0.9 and noise overlap (0.05)) and further manually curated based on clean spike shapes and autocorrelograms with clear refractory periods. To assess firing rates (Supplementary Fig. 2f), neurons with >10 spikes during center arm travel (*n* = 92 units) were included. To measure within-day consistency, neurons with >100 spikes during the entire recording session were considered (*n* = 82 units). Spatial tuning functions were obtained by binning the spike count as a function of linearized position during center and side arm travel. Consistency was obtained by Pearson’s r between tuning functions of odd vs. even trials and averaged over all neurons and sessions per mouse (Supplementary Fig. 2g). The analysis was performed on data downsampled to 1 kHz.

### Histological processing

Mice were deeply anesthetized by intraperitoneal injection of ketamine/xylazine (100/13 mg/kg body weight). After cessation of pain reflexes, the thorax was opened to expose the heart, and an incision was made into the right atrium. Perfusion commenced into the left ventricle with ice-cold PBS (~5 ml), followed by 4% paraformaldehyde (PFA, ~15–20 ml). The brains were removed from the skull and post-fixed in 4% PFA at 4 °C overnight for retrograde tracing experiments or for >2 days for removing the GRIN lens.

Coronal sections were cut with a vibratome (Leica VT1000S, 100 µm thickness), stained with 4′−6-diamidino-2-phenylindole (DAPI, 1:1000 in PBS), and mounted on microscope slides with Mowiol. Confocal image stacks were obtained with a confocal laser-scanning microscope (Zeiss LSM 710 or 900).

### Immunohistochemistry

To assess number of microglia, level of phagocytic state of microglia, and number of astrocytes, immunohistochemistry against Iba1 (wako pure chemicals 019-19741), CD68 (Abcam ab125212) and GFAP (Abcam ab4674), respectively, was performed. Fixated (in 4% PFA) brain slices were permeabilized in PBS + 0.4% TritonX-100 for two times each 30 min. Slices were then blocked in PBS + 0.2% TritonX-100 + 4% Normal Goat Serum (NGS) for 30 min. Afterwards, slices were kept at room temperature for 6 h in PBS + 0.1% TritonX-100 + 2% NGS and the respective primary antibody (GFAP 1:500, Iba1 1:1000, CD68 1:500). Slices were further incubated overnight at 4 °C. Next day, slices were washed in PBS + 1% NGS, which was repeated three times for 10 min each. Slices were then incubated with the secondary antibody (goat anti-chicken Alexa Fluor® 647 (ab150171) or goat anti-rabbit Alexa Fluor® 647 (ab150079)) for 2.5 h. Slices were then washed two times in PBS + 1% NGS for each 10 min. Finally, slices were stained with DAPI (1:1000 in PBS) for 5 min and thereafter washed with PBS for three times each 10 min. Except for the overnight incubation all steps were performed at room temperature.

For quantification of the staining, ImageJ (V1.52p) was used. Two areas, either underneath the implanted lens or on the contralateral side were drawn and the mean intensity within these areas were measured.

### Extraction of calcium signals

We used the open-source Python toolbox *CaImAn*^45^ to identify the neurons’ shape (spatial component) and the corresponding calcium trace (temporal component, Supplementary Fig. 3a). For motion correction, a spatial high-pass filter (Gaussian kernel size 10 pixel; 7.5 µm) was applied on all imaging frames to obtain sharp images. Rigid shifts were then inferred by maximizing the cross-correlation between each frame and a template image that was updated by taking the median image of the motion-corrected movie. Next, source extraction was achieved using the ‘GreedyCorr’ method in CaImAn that implemented the CNMF-E algorithm to remove background light signals from the cellular light sources in 1-photon recording^46^. The algorithm was initialized with the parameters: ‘min_corr’ (minimum correlation) and ‘min_pnr’ (minimum peak-to-noise ratio), chosen for each mouse in cohort 1 (listed in Table 1). For mice in cohort 2 (learning experiment), ‘min_corr’ and ‘min_pnr’ were determined using the Otsu’s thresholding method on the correlation and the peak-to-noise ratio image (threshold means as listed in Table 1). The quality of extracted components was quantified by the correlation value of each spatial component with the frames where this component was active (rval) and the signal-to-noise ratio (SNR). Components with rval >0.7 or SNR > 2 were kept for further inspection. Lastly, we used a custom GUI to inspect components and classify them as putative cells (https://github.com/chenhungling/CaimanGUI). Components with infiltrating calcium activity from neighboring components as well as cells with ambiguous shape or calcium traces were discarded.

**Table 1:** CaImAn parameters used to extract calcium signals

	mouse	min_corr	sem	min_pnr	sem
cohort 1	#44	0.7	-	4	-
	#45	0.7	-	5	-
	#47	0.7	-	5	-
	#93	0.7	-	6	-
	#94	0.8	-	4	-
	#95	0.8	-	6	-
	#216	0.8	-	4.5	-
	#218	0.8	-	6	-
	#219	0.8	-	3.5	-
cohort 2	#478	0.787	0.001	12.718	0.099
	#480	0.780	0.004	10.760	0.197
	#481	0.798	0.003	12.290	0.081
	#483	0.783	0.001	12.097	0.129
	#485	0.813	0.001	14.525	0.101

Min_corr and min_pnr describe minimal thresholds for the initialization of component identification, namely the minimal correlation value and minimal PNR.

Calcium traces were further corrected for slow drifts of the baseline using a running percentile filter (10th percentile, window size 30 s). Then, the traces were standardized by iteratively calculating the mean (baseline) and standard deviation (σ). During each iteration, the signal above 3σ was excluded until the relative change in σ was smaller than 0.1% ((σ_0_–σ_1_)/σ_1_ < 0.001). The baseline-subtracted and normalized trace was used for the analysis unless indicated otherwise (referred to as ‘z-scored traces’). For the additional assessment of calcium transients, significant transients were calculated based on the z-scored traces as signals exceeding 3σ and lasting for a minimum duration of 0.2 s. The rising phase was obtained from these transients by taking for each transient the signal from threshold crossing to the first peak after the threshold.

### Longitudinal registration of active neurons

For longitudinal registration, the data of cohort 1 were split into two blocks. The first one included data of the first 24 recording days with a consistent rule (Figs. 1, 2, and 3). The second block included the data over rule switches (Fig. 6). Data from cohort 2 (learning experiment, Fig. 4) were analyzed in a single block. The *CellReg*^47^ algorithm was used to identify the same cells in different imaging sessions (Supplementary Fig. 3). First, the projection images of all cells per session were aligned across sessions by maximizing the image cross-correlation with a reference session (cohort 1) or using an optical flow-based method (*skimage.registration.optical_flow_ilk*, cohort 2, Supplementary Fig. 3). Then, the data of the spatial correlation between each cell-pair across different sessions (defined by a centroid distance <31 ± 1 pixel (23.3 ± 0.08 µm)) was collected (Supplementary Fig. 3d shows an example of data distribution of spatial correlation). The data was fitted by a weighted sum of two distributions: a lognormal distribution for modeling ‘same’ cells and a beta distribution for modeling ‘different’ cells. The best fit for each mouse and respective CellReg block were found by minimizing the mean squared error (MSE; first block: 0.12 ± 0.01) between the collected data and the model. Each cell pair across sessions was then characterized by a probability to be the same cell (*P*_same_). Finally, cells were registered via an iterative clustering procedure where each cell either formed a new cluster if no *P*_same_ > 0.95 was found, or assigned to the cluster with the highest *P*_same_. We obtained an average true positive score (first block: 0.971 ± 0.004) and true negative score (0.944 ± 0.007).

Unless indicated otherwise, we considered only neurons that were detected as active on each day of the registration set. For analyses during the execution of the original rule in the first block, registration was done over 24 recording days. For continuous and pause assessment, 2 sessions that were 7 recording days away from each other were used for registration. Registration was performed over 2 days to measure familiar versus novel (*n* = 8 mice) and 9–11 recording days (mean: 10 ± 0.26, *n* = 6 mice) to assess original, new, and restored conditions. For initial rule learning in cohort 2, registration was performed separately over 2 recording days 4 days apart from each other during learning and two equally spaced recording days after reaching criterion (Fig. 4). Due to uneven sampling of the task space in some mice of the before learning group, only left-going trajectories were analyzed in this experiment.

### Extraction of spatial positions and maze areas

Tracking of the center point of the animal’s position was performed by EthoVision XT 11.5 (Noldus). The animal’s position in the maze (behavioral epochs: sampling, left/right center, left/right side, left/right reward) was identified by xy-coordinate thresholds using a custom written python-script. Only correct trials were used for further analysis.

### Linearization of 2d trajectories

We mapped 2D trajectories onto a 1D skeleton, which approximated the linear position as a series of 4 vectors (Supplementary Fig. 5f). For each x/y coordinate, the nearest point on the skeleton was found using the *scipy.spatial.KDTree* function. The linear position from entry into the center arm to the end of the side arm was then expressed as normalized distance (from 0 to 1). To assess the point of deviation of trajectories during left and right trials in the center arm, each left and right run was linearized in 1D and interpolated by a factor of 10, giving the linear position of the run. Similarly, the x-coordinate of each run was interpolated by a factor of 10, giving the x-position. X-positions were then binned as a function of linearized position (*n* = 50 bins). For each bin, the x-position during left and right trajectories were statistically compared using an independent t-test (with Bonferroni correction for 50 comparisons). The first bin with a significant difference was taken as the point of trajectory divergence.

### Correlation analysis

Spatial tuning functions were obtained by first binning z-scored calcium signals as a function of linearized position during outward travel (20 bins, covering center and side arm). The binned signals were divided by the occupation probability of the bins and normalized to the bin with the largest mean activity. To obtain spatial consistency within a day, the tuning functions of each neuron were computed separately for odd and even runs during left and right trials. Pearson’s r for odd vs. even runs of the same direction (i.e., left vs. left and right vs. right) or across directions (i.e., left vs. right and right vs. left) was used to assess the similarity of spatial trajectories. Correlation values were averaged over cells for each mouse to obtain one value per animal. To obtain the correlation of tuning functions across days, Pearson’s r was computed for the average tuning function of each neuron on day 1 versus the other days, separately for left and right trajectories. The values were then averaged over left and right conditions and neurons to get one correlation value per mouse and day. To get random control data for comparison, shuffled data were created by randomly permuting the order of the tuning functions of day 1 such that correlation over days was performed between different neurons (average of 100 permutations). The same analysis was applied to learning vs. learned, continuous vs. pause, familiar vs. novel, original vs. new, and original vs. restored comparisons. In addition, we compared the across-days correlations of the real data to surrogate data with simulated representational drift. Separately for the left- and right-going normalized tuning functions of each neuron, we iterated over the probability for the tuning function to circularly drift by 1 bin over 1 recording session (drift probability: 0–100%, step size 10%). An additional noise term taken from a normal distribution (mean: 0, SD ranging from 0 to 0.5) was added to the tuning functions. On each successive session, the neurons with and without drift between sessions were randomly and independently drawn according to the drift probability. To avoid tuning functions to drift back towards the original tuning function (which spans 20 bins), the drift function was regularized such that a cumulative drift >10 bins results in a random drift of −1 or 1 bin on the next drift step.

### Mean activity and side index

Mean activity was defined as the mean signal of significant calcium transients during center or side arm travel. Side index *S* of each neuron was defined as

1 $$S=\frac{{a}_{l}-{a}_{r}}{{a}_{l}+{a}_{r}}$$

where a_l_ and a_r_ are the mean signals of significant calcium transients of the neuron during left and right trajectories, respectively. For the display in Fig. 1 and Supplementary Fig. 10c, d repeatedly active neurons with a minimum mean activity of 0.5 z were considered (*n* = 133 and 19 neurons, respectively). However, different activity thresholds gave reproducible results for the correlation of mean activity and side index over time (thresholds: 0.01, 0.05, and 0.1–1.0, 0.1 increments, Supplementary Fig. 4), and for the dependence of decoding accuracy of single neurons on absolute side score (thresholds: 0.2–1.0, 0.1 increments, Supplementary Fig. 6b).

### Spatial information

Spatial information^6^ was obtained from significant calcium transients of repeatedly active neurons separately for left and right trials as

2 $${SI}=\mathop{\sum}\limits_{i=1}^{N}{\lambda }_{i}{{{{\mathrm{ln}}}}}\frac{{\lambda }_{i}}{\lambda }{p}_{i}$$

where *λ*_*i*_ is the mean activity of the neuron in the ith bin, λ is the average activity across the trajectory, *p*_*i*_ is the occupation probability of the ith bin, and *N* is the number of bins of the outward trajectory (*N* = 20).

### Decoder analysis

Two decoders were used to predict position and trial outcome across days (see below). In general, the models were trained on the first recording day for each comparison. For the analysis over 24 d in the first block, all available neurons that were active on each of the recording days were used (502 total, 6–131 per mouse, mean: 63 ± 16, *n* = 8 mice). To assess continuous and pause pairs, neurons active during both days of each pair were used (*n* = 958 total, 20–266 per mouse, mean: 120 ± 31, and *n* = 945 total, 21–235 per mouse, 118 ± 32, *n* = 8 mice). In the second block, decoding was performed for each mouse using all neurons that were active on the last day of the original rule and on the first days of both new and restored rules, respectively (272 total, 11–120 neurons per mouse, mean: 45 ± 17, *n* = 6 mice). To assess familiar and novel conditions, all neurons active on two consecutive days in the familiar arena (*n* = 1261 total, 54–248 per mouse, mean: 157 ± 25, *n* = 8 mice) or during the last day in the familiar and the first or second day in the novel arena (*n* = 1267 total, 45–269 per mouse, mean: 158 ± 27, *n* = 8 mice) were used. To compare before learning and learned groups, the models were trained and tested on 20 separate iterations during which 50 neurons were randomly selected each time per mouse. Since four mice of the before learning group showed insufficient rightward trials, the before learning-learned comparison was done on leftward trajectories only.

### Decoding of spatial position

Position decoding was performed using support vector regression with the *scikit-learn*^48^ package in Python3.7.7 (*sklearn.svm.LinearSVR*, parameters: C = 1, max_iter = 100000). Linearized position during outward travel was decoded from z-scored calcium signals. Alternatively, calcium transients and rising phases of transients were used as indicated. All left runs (and separately all right runs) of the first day were used to train the decoder, and all runs of the same direction on the target day were used for decoding. Results for left and right runs were averaged. To assess the impact of SI, the data of each mouse was split in the 50% of neurons with largest and lowest SI, and decoding was run separately on both sets. Decoding accuracy was quantified as the mean absolute error using *sklearn.metrics.mean_absolute_error* (ranging from 0 to 1). Shuffled data were created by permuting the position data in the training dataset (separately for left and right trials, 100 iterations).

### Decoding of trial outcome

Logistic regression was implemented with *sklearn.linear_model.LogisticRegression* (parameters: C = 5, penalty = ’l2’, max_iter = 1000). Within each day, the data were sorted by trials, and z-scored calcium signals (or significant transients or rising phases where indicated) were averaged for each cell and trial during the center epoch. To decode across days, all trials of the first day served as the training dataset, and decoding was done on all trials of the target day. Shuffled control data were obtained by averaging the decoding results on permuted trial labels of the target day (100 iterations). To assess decoding during the period before diversion of trajectories for left and right trials within a day, the z-scored calcium signals for each cell were averaged until the diversion point for each trial. Next, decoding was performed in a leave-one-out regime such that the outcome of a randomly selected trial was predicted using the remaining trials as training data. This procedure was repeated 100 times with a randomly selected test trial, and the accuracy of the decoding was expressed as the proportion of correctly predicted trials. To obtain shuffled control data, the trial labels were permuted independently during each iteration, and the random prediction was averaged. To resolve the number of neurons needed for decoding, a random subset of neurons of increasing size was drawn from the total population, and the decoding was performed as before.

To test the contribution of individual neurons to trial outcome decoding, decoding was performed separately for each cell in the first block. To remove neurons without activity in the selected task period, a cut-off for the minimal required calcium activity was set at 0.5 z (leaving the model with 133 neurons from 8 mice in total), but different threshold values gave comparable results (Supplementary Fig. 6b). Decoding accuracy was measured by applying a leave-one-out strategy such that the model was trained on the z-scored calcium activity of one neuron during all but one trial of a given day and then used to predict the outcome of the remaining trial. This was repeated for all trials of each day. Spearman’s correlation coefficient was used to assess the correlation between decoding accuracy of the neurons on day 1 and 24.

To test whether trial outcome decoding depends on the chosen model, the above analysis based on all available neurons was repeated with a support vector classifier (*sklearn.svm.SVC*, parameters: C = 5, penalty = ’l2’, max_iter = 1000) and with an artificial neural network (Supplementary Fig. 5e). The latter was implemented in *pytorch* as a 3-layer network with hidden sizes = 200 neurons and ReLu non-linear activation functions. The network was trained on data from day 1 in 500 epochs (learning rate: 0.0001) to minimize the *BCEWithLogitsLoss* function. As shuffled controls for this analysis, the calcium data of each neuron was randomly shifted in time (5–20 s) before training. The trained models were used to make binary predictions based on calcium data of the following recording days.

### Generalized linear modeling

To identify encoding of linearized position, speed, and goal location in the imaged cell population we used a GLM with a linear link function^14^. The z-scored calcium signal was concatenated for correct, outward trials (i.e., from sampling to reward). Similarly, speed and linearized position were concatenated to match the calcium signal. Left or right goal location was indicated with 1 or 2 for the entire trial, respectively. Position was binned in 50 equal bins. The first and last bin were kept (sampling and reward location), the others were added to create 8 bins across the trajectory (smaller bin sizes were tested and did not show any difference, similar to previous reports^14^). Speed was categorized in quartiles, matching the speed of the individual animal. All predictors described above were used as categorical variables. The ‘full model’ contained all predictors, the ‘single-models’ only one selected predictor, and the ‘shifted models’ all predictors, but one randomly shifted in time to not match the neuronal data anymore. These linear models were then fitted (Matlab function *fitglm*) on 90% of (training) data points and used to predict the remaining 10% (test) data points. To diminish training and testing on directly adjacent events, data was chunked in 5 s bins, which were then randomly assigned to training and testing datasets. Models were 10 times cross-validated, to ensure all data was used for training and testing.

Explained variance was determined by the squared correlation (Pearson’s *r*; *r*^2^) of predicted and actual calcium data. The decrease in explained variance (Δ*r*^2^), when one predictor was shifted, was calculated as the difference in explained variance of the full model and the shifted model. To identify whether individual cells were modulated by a specific predictor, the difference between the *r*^2^ of the ‘full model’ and the *r*^2^ of a model without the respective predictor (predictor was set to zero) was calculated. This Δ*r*^2^_without_ was compared to a Δ*r*^2^_shuffled_-distribution (generated by calculating the difference in *r*^2^ between a model with the predictor shifted and a model where the predictor was set to zero, 1000 times). If the cell’s Δ*r*^2^_without_ was greater than 99% of the Δ*r*^2^_shuffled_-values, the neuron was considered to be significantly modulated by that variable.

### Geometry of the population response

We used a geometrical approach to analyze population vector activity^3^. Population activity as a function of space was extracted from z-scored calcium activity of all *n* repeatedly active neurons of a recording session during three spatial bins: At the start of the outward trajectory, the midpoint, and the endpoint, separately for left and right trajectories (bins correspond to the same 20 bins as used for the construction of spatial tuning functions). We constructed the geometrical object with corner points given in *n* dimensions by each population vector, and extracted the edge angles between vectors connecting the corner points (10 edge angles for each of the 6 corner points). The dissimilarity between the resulting edge angle matrix of each day with day 1 was expressed as the edge angle similarity matrix ||*A*^*a,b*^|*|*_*F*_ using the Frobenius norm

3 $${{{{{{\rm{||}}}}}}{A}^{a,b}{{{{{\rm{||}}}}}}}_{F}=\sqrt{{\sum }_{i=1}^{M}{\sum }_{j=1}^{N}{|{A}_{i,j}^{a}-{A}_{i,j}^{b}|}^{2}}$$

where A^a^ and A^b^ denote edge angle matrices on day *a* and *b*, *M* denotes the number of corner points ( = 6), and *N* denotes the number of angles of incoming pairs of edges from other corner points ( = 10). ||*A*^*a,b*^|*|*_*F*_ was computed separately for odd and even runs and averaged for each session. This allowed normalization using the same odd-even matrices of each day to estimate within-day dissimilarity. Normalization was applied to ||*A*^*a,b*^|*|*_*F*_ to obtain the normalized edge angle dissimilarity ||Â^*a,b*^|*|*_*F*_ as

4 $${{{{{{\rm{||}}}}}}{A}^{a,b \,\wedge}{{{{{\rm{||}}}}}}}_{F}=\frac{{{||}{A}^{a,b}{||}}_{F}-{{{{{{\rm{||}}}}}}{A}^{w}{{{{{\rm{||}}}}}}}_{F}}{{{{{{{\rm{||}}}}}}{A}^{s}{{{{{\rm{||}}}}}}}_{F}-{{{{{{\rm{||}}}}}}{A}^{w}{{{{{\rm{||}}}}}}}_{F}}$$

where ||A^W^||_F_ is the within-day Frobenius norm computed between odd and even runs averaged over all days, and ||A^S^||_F_ is the Frobenius norm between matrices of the last and first day obtained after column- and row-shuffling the tuning functions. The resulting metric is thus bound between 0 (equivalent to the dissimilarity over time being equal to the within-day dissimilarity) and 1 (with the dissimilarity being equal to the dissimilarity of shuffled data). Population vectors were obtained from the same three spatial points as the average of the significant transients of each neuron, and correlated with day 1 using Pearson’s r.

### Statistical analysis

Comparisons between 2 dependent groups (e.g., left and right trials or data and shuffled datasets from the same animal) were made with two-sided paired t-tests (for normally distributed data, assessed by a Shapiro–Wilk test) or Wilcoxon signed-rank tests (for data that did not follow a normal distribution). For comparisons of independent data, unpaired two-sided *t*-tests were used for normally distributed data, and Mann–Whitney *U*-test for data that did not follow a normal distribution. For comparisons of multiple time points with a single group (e.g., behavioral performance over days), one-way repeated measures ANOVA was used. To compare time series of two groups (e.g., spatial correlation to day 1 for data and shuffled controls), two-way repeated measures ANOVA was used. Pairwise comparisons commenced for each time point using paired t-tests with Šidák correction. Unless indicated otherwise, statistics were based on averages per mouse. Statistical tests were computed using Python’s *stats* and *pingouin*^49^ packages. Data are expressed as mean ± sem.

### Reporting summary

Further information on research design is available in the Nature Portfolio Reporting Summary linked to this article.

### Supplementary information

Supplementary Information

Peer Review File

Description of Additional Supplementary Files

Supplementary Movie 1

Reporting Summary

### Source data

Source Data

## Data Availability

The calcium and behavioral data generated in this study have been deposited in the Zenodo database under accession code 10.5281/zenodo.10528243. Source data are provided with this paper.

## Code availability

Custom code is available under accession code 10.5281/zenodo.10528243.
