# Supplementary material for: A persistent prefrontal reference frame across time and task rules
Source: Nat Commun. 2024 Mar 8;15:2115. doi: 10.1038/s41467-024-46350-4 (PMC10923947; doi:10.1038/s41467-024-46350-4)
Supplement: Supplementary file 1 — Supplementary Information [file 41467_2024_46350_MOESM1_ESM.pdf]

A persistent prefrontal reference frame across time and task rules

Hannah Muysers, Hung-Ling Chen, Johannes Hahn, Shani Folschweiller, Torfi Sigurdsson, Jonas-Frederic Sauer*, Marlene Bartos*

Supplementary Materials

Supplementary Figures 1-10, Supplementary Movie 1

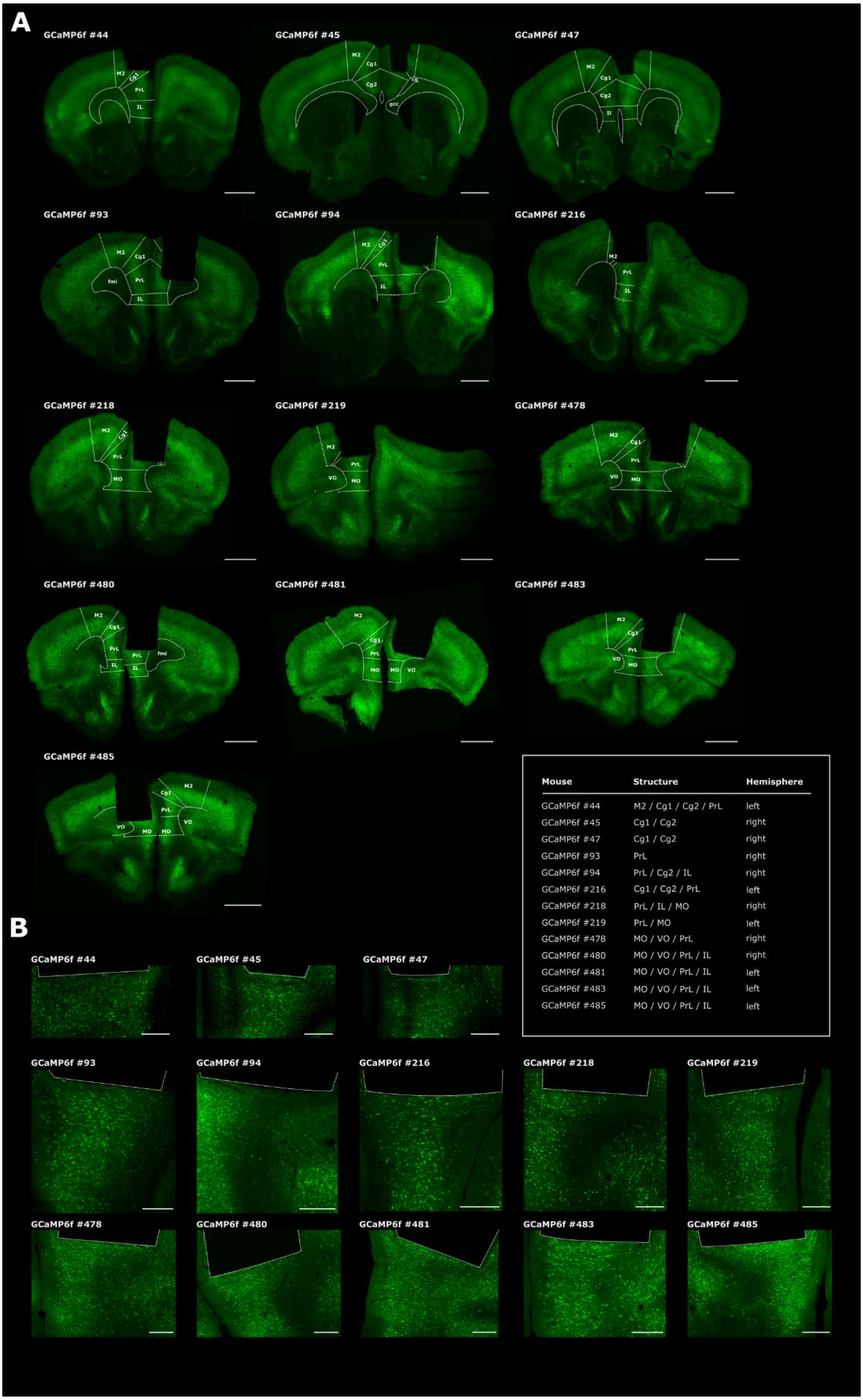
A
GCaMP6f #44
GCaMP6f #45
GCaMP6f #47
GCaMP6f #93
GCaMP6f #94
GCaMP6f #216
GCaMP6f #218
GCaMP6f #219
GCaMP6f #478
GCaMP6f #480
GCaMP6f #481
GCaMP6f #483
GCaMP6f #485
Mouse Structure Hemisphere
GCaMP6f #44 M2 / Cg1 / Cg2 / PrL left
GCaMP6f #45 Cg1 / Cg2 right
GCaMP6f #47 Cg1 / Cg2 right
GCaMP6f #93 PrL right
GCaMP6f #94 PrL / Cg2 / IL right
GCaMP6f #216 Cg1 / Cg2 / PrL left
GCaMP6f #218 PrL / IL / MO right
GCaMP6f #219 PrL / MO left
GCaMP6f #478 MO / VO / PrL right
GCaMP6f #480 MO / VO / PrL / IL right
GCaMP6f #481 MO / VO / PrL / IL left
GCaMP6f #483 MO / VO / PrL / IL left
GCaMP6f #485 MO / VO / PrL / IL left
B
GCaMP6f #44
GCaMP6f #45
GCaMP6f #47
GCaMP6f #93
GCaMP6f #94
GCaMP6f #216
GCaMP6f #218
GCaMP6f #219
GCaMP6f #478
GCaMP6f #480
GCaMP6f #481
GCaMP6f #483
GCaMP6f #485

Supplementary Fig. 1: Targeting the mPFC for calcium imaging.

a) Confocal images showing the position of the lenses (0.5 (GCaMP6f #45, #47) or 1 mm diameter) used for imaging from the mPFC. Prelimbic and cingulate cortex were targeted for recording. Green: GCaMP6f. Scale bar is 1 mm. (M2: secondary motor cortex, Cg1: cingulate cortex area 1, Cg2: cingulate cortex area 2, PrL: prelimbic cortex, IL: infralimbic cortex, MO: medial orbital cortex, VO: ventral orbital cortex)

b) Higher magnification showing tissue underneath the lens. Focus is 200-300μm below the lens. Scale bar is 250 μm.

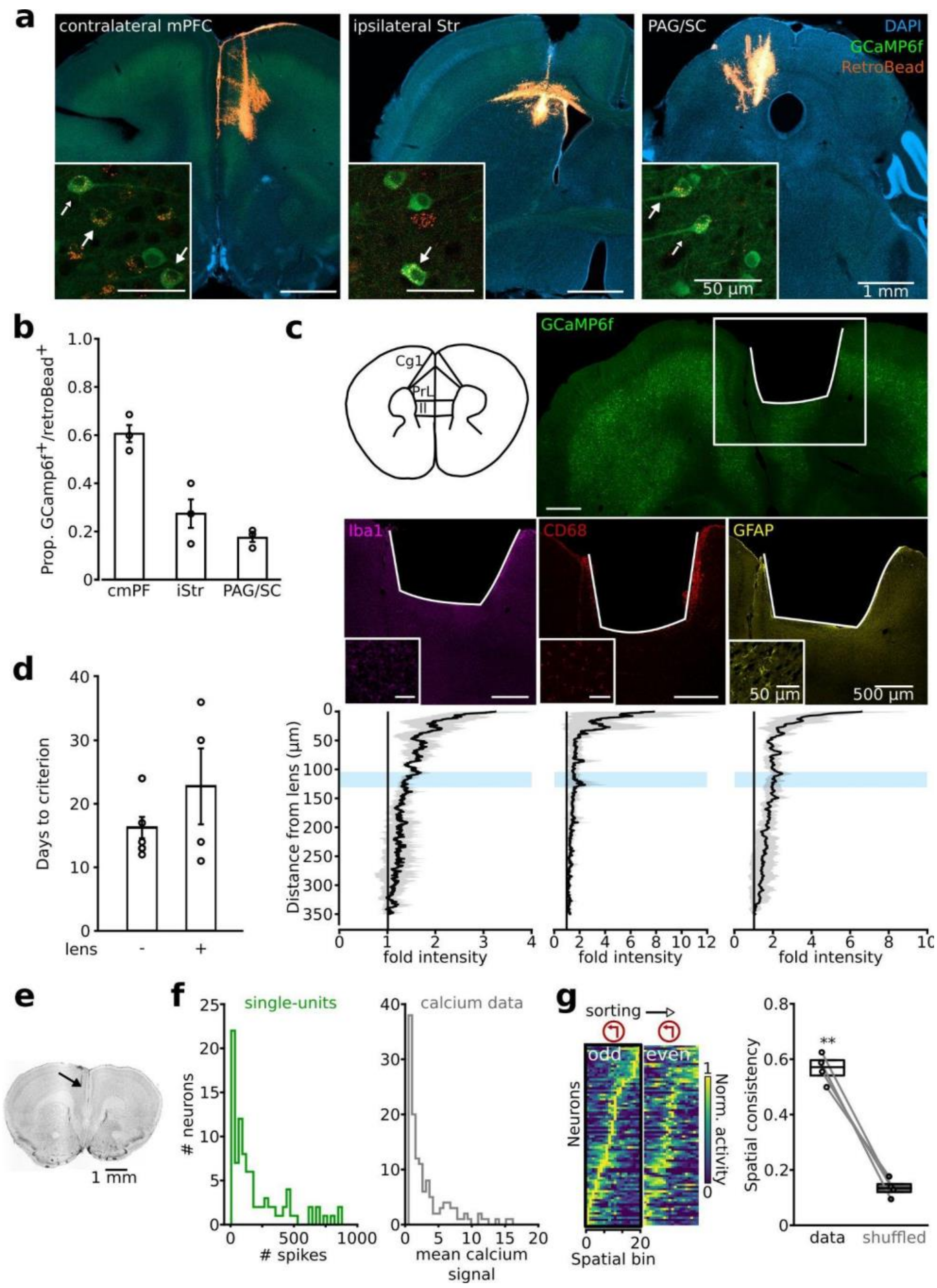

Supplementary Fig. 2: Characterization of imaged cell types and effect of lens implantation on histology and learning.

a) Retrograde labeling with redRetroBead was used to assess distinct mPFC projection neurons. Left: retroBead injection into the contralateral mPFC (cmPFC) labels GCaMP6f-positive neurons in the ipsilateral hemisphere (inset), indicating that intratelencephalic (IT) neurons are present in the recorded population of GCaMP6f-positive neurons. Middle: Injection into the periaqueductal gray (PAG)/superior colliculus (SC) labels GCaMP6f-positive neurons in the mPFC (inset), indicating that pyramidal tract (PT) neurons are present in the recorded population of GCaMP6f-positive neurons. Right: Injection into ipsilateral striatum (iStr), a projection target of both IT and PT neurons, reveals double-labeled GCaMP6f-positive neurons in the mPFC.

b) Quantification of the proportion of GCaMP6f-positive neurons co-labelled with retroBead for the 3 injections side shown in a). cmPFC: 962 GCaMP6f^{+}/retroBead^{+} and 596 GCaMP6f^{+}/retroBead^{-}, PAG/SC: 100

GCaMP6f$^+$/retroBead$^+$ and 435 GCaMP6f$^+$/retroBead$^-$, iStr: 443 GCaMP6f$^+$/retroBead$^+$ and 1229 GCaMP6f$^+$/retroBead$^-$, n=3 mice each. Dots show averages of 2-3 sections per mouse.

c) Top: GCaMP6f labeling and lens implantation side. Middle: Staining for Iba1 (purple), CD68 (red) and GFAP (yellow) to reveal the number of microglia, level of phagocytic state of microglia, and number of astrocytes, respectively, at the imaging location. Bottom: Quantification of labeling intensity as a function of distance from lens edge. The imaging plane (blue box) is 114±8µm below the lens surface. Data are shown as fold intensity compared to the contralateral (unimplanted) side. n=3 mice.

d) Lens implantation does not affect task learning. Bars show the means of cohorts trained in the task before (‘without GRIN lens’, n=8) and after lens implantation (‘GRIN lens’, n=4) to reach the learning criterion (≥70% correct on three consecutive sessions), dots show the same for single mice. One-sided Mann–Whitney U=11.5, p=0.246.

e) Visualization of tetrode recording sites (arrow) in the mPFC.

f) Similar distribution of firing rates (green, spike numbers, n=93 units from 4 mice) and mean calcium signal intensity (gray, n=133 neurons with mean activity >0.5 as shown in **Fig. 1**) from electrophysiological and 1-photon imaging experiments, respectively, during task execution.

g) Single-unit recordings identify large within-session stability of spatial tuning. Left: Tuning functions (n=82 neurons from 4 mice) during left-going odd and even runs sorted for odd runs. Right: Mouse averages (white) of spatial consistency vs. cell ID-shuffled data (gray). T=10.51, p=0.0018, 2-sided paired t-test.

Bars show mean ± sem, boxes show median and upper/lower quartiles. Source data are provided as a Source Data file.

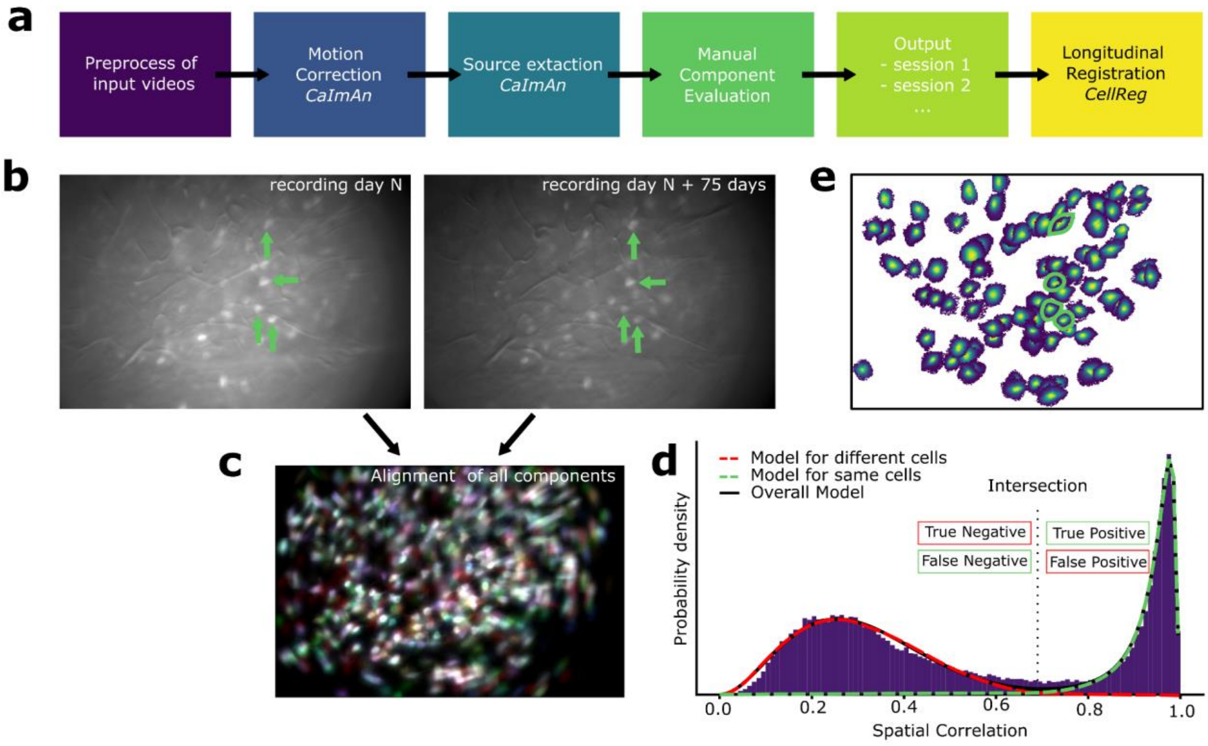

Supplementary Fig. 3: Experimental pipeline for multi-session registration.

a) Workflow of data analysis: Movies were first pre-processed and motion corrected. Potential cells were identified with CaImAn[45]. All neurons were then checked for infiltrating calcium activity of neighboring cells and ambiguous shape of the cell or its calcium signals. The output was stored per session and CellReg[47] was used to longitudinally identify the same set of cells over days.

b) Two field of views (FOVs; projection across entire recording in olfaction guided memory task) from the same mouse with 75 days in between the two recording sessions. Green arrows point to the same cells in both FOVs.

c) Alignment of 16 sessions and overlay of the identified components in each session (different color coding). Cells which are identified in multiple sessions appear as white.

d) Distribution of the spatial correlation of cell-pairs (radius of 22.5 μm) across sessions. The data is fitted with a weighted sum of two distributions: Green for same-cell model, red for different-cell model. The vertical dashed line indicates the intersection of the two models.

e) Example of all cells which are active and identified in all 16 recording sessions.

c), d) and e) are based on the CellReg algorithm[47].

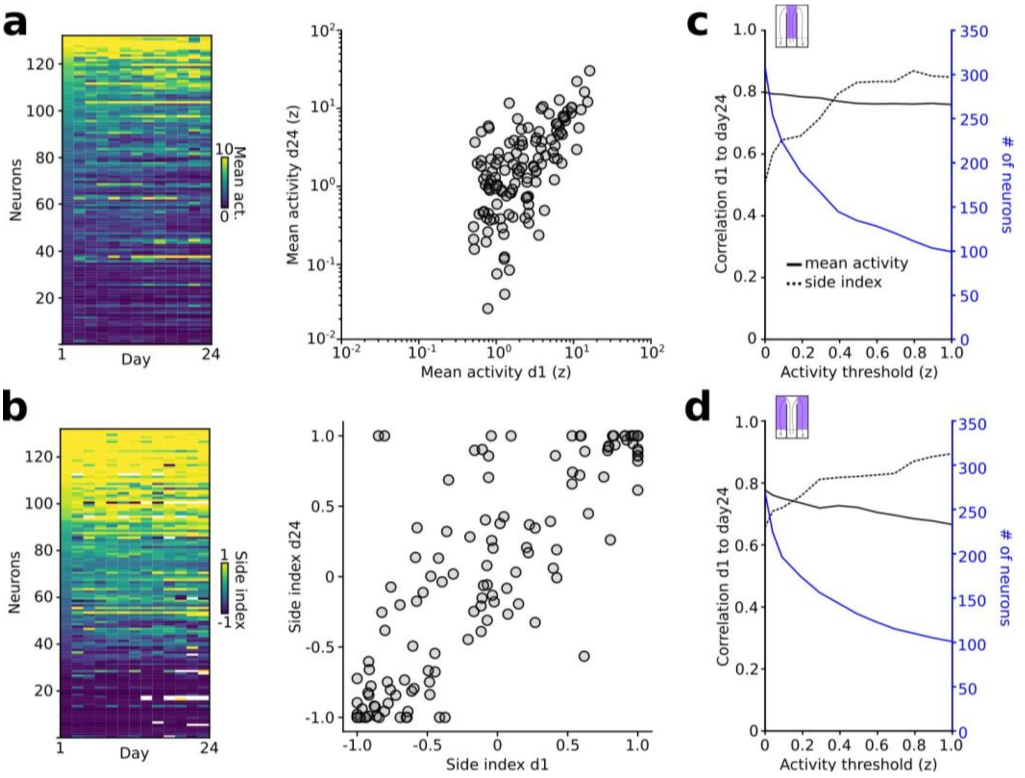

Supplementary Fig. 4: Stable responses during side arm travel

a) Stable mean calcium activity during side arm travel over days. The plot on the right shows mean activity during the first and last day (Spearman's r = 0.754, p=2*10^{-23}, n=133 neurons). 2-sided correlation tests.

b) Stable preference for activity during left or right trials (side index). The plot on the right shows side index during the first and last day (Spearman's r = 0.823, p=2*10^{-32}, n=133 neurons). 2-sided correlation tests.

c) Correlation (day 1 to 24) of mean activity (solid black line) and side index (dashed black line) in the center arm for different thresholds of required activity (0.0-1 z) along with the number of repeatedly active neurons available at each threshold level (blue).

d) Same as c) but for side arm travel.

Source data are provided as a Source Data file.

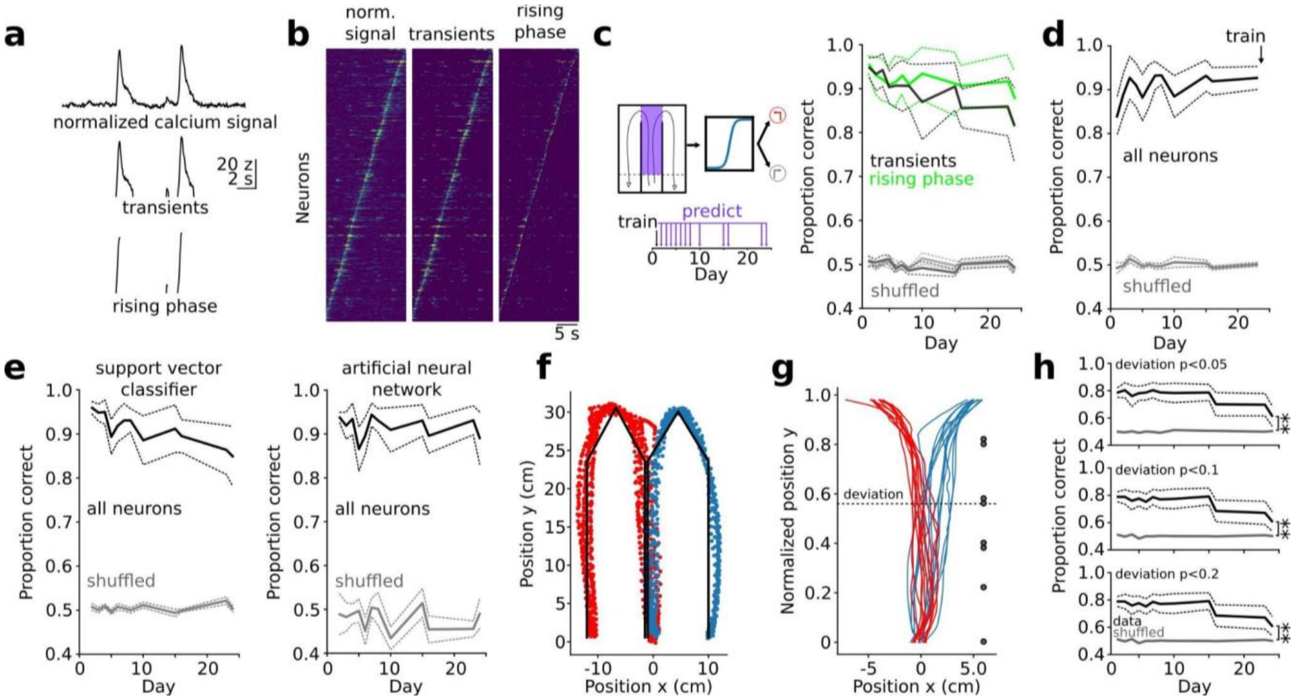

Supplementary Fig. 5: Additional analysis of trial outcome decoding.

a) Example of normalized calcium signal as used in the main figures (top), extracted significant transients (i.e., exceeding 3 SD of the normalized signal, middle) and rising phases of calcium transients used for decoding.

b) Spatial maps of the three signal types on the first recording day (n=1109 neurons). Maps are shown with the same sorting.

c) Decoding trial outcome with transients (black) and rising phases (green). Decoding performance significantly exceeded shuffled data for both transients (F=74.20, p=6*10^{-5}) and rising phases (F=76.71, p=5*10^{-5}), with no time-dependent decay (decoding accuracy-time interaction effect: transients: F=1.333, p=0.289, rising phases: F=0.65, p=0.634), n=8 mice. Shuffled data in light gray are for transients, in dark gray for rising phases.

d) Trial outcome can be decoded over previous days with a model trained on day 24. F=123.34, p=10^{-6} versus random. Effect of time: F=1.57, p=0.125. Time*condition interaction: F=1.15, p=0.333, n=8 mice.

e) Decoding over time using different models trained on day 1. Effect of performance versus shuffled data: F=170.58, p=10^{-6}; F=135.06, p=10^{-6}. Effect of time: F=1.20, p=0.300; F=1.22, p=0.291. Time*condition interaction: F=1.54, p=0.133; F=0.46, p=0.921, n=8 mice.

f) Illustration of the linearization of the 2-D track in 1-D. Shown are all left (red) and right (blue) trials of a mouse. Black line indicates the skeleton on which the x and y coordinates were projected.

g) Determination of significant diversion between left and right trajectories during center arm travel. The dotted line shows the first position for which trajectories deviated significantly (p<0.05) from each other. Points on the right show the position of deviation for all 8 mice in the task.

h) Trial outcome can be significantly predicted over time from average calcium signals before deviation of left and right trajectories. A logistic regression model was trained on day 1 to predict trial outcome on successive days. Top: main effect of decoding performance versus shuffled data (F=28.30, p=0.002) but no effect of time

(F=1.60, p=0.117) or time*condition interaction (F=1.69, p=0.095). N=7 mice (one mouse was not included in this analysis as it showed deviations already at the sampling point, presumably reflecting immediate choice as reported previously in rats[21]). Middle/bottom: Across-day decoding was robust against a different choice of threshold for the detection of the trajectory deviation point (p<0.1: effect of performance: F=24.28, p=0.003, effect of time: F=1.94, p=0.0496, effect of time*condition interaction: F=1.81, p=0.070; p<0.02: effect of performance: F=22.89, p=0.003, effect of time: F=1.75, p=0.080, effect of time*condition interaction: F=1.48, p=0.159).

Continuous and dotted lines indicate mean ± sem. All statistical comparisons: 2-way repeated measures ANOVA. Source data are provided as a Source Data file.

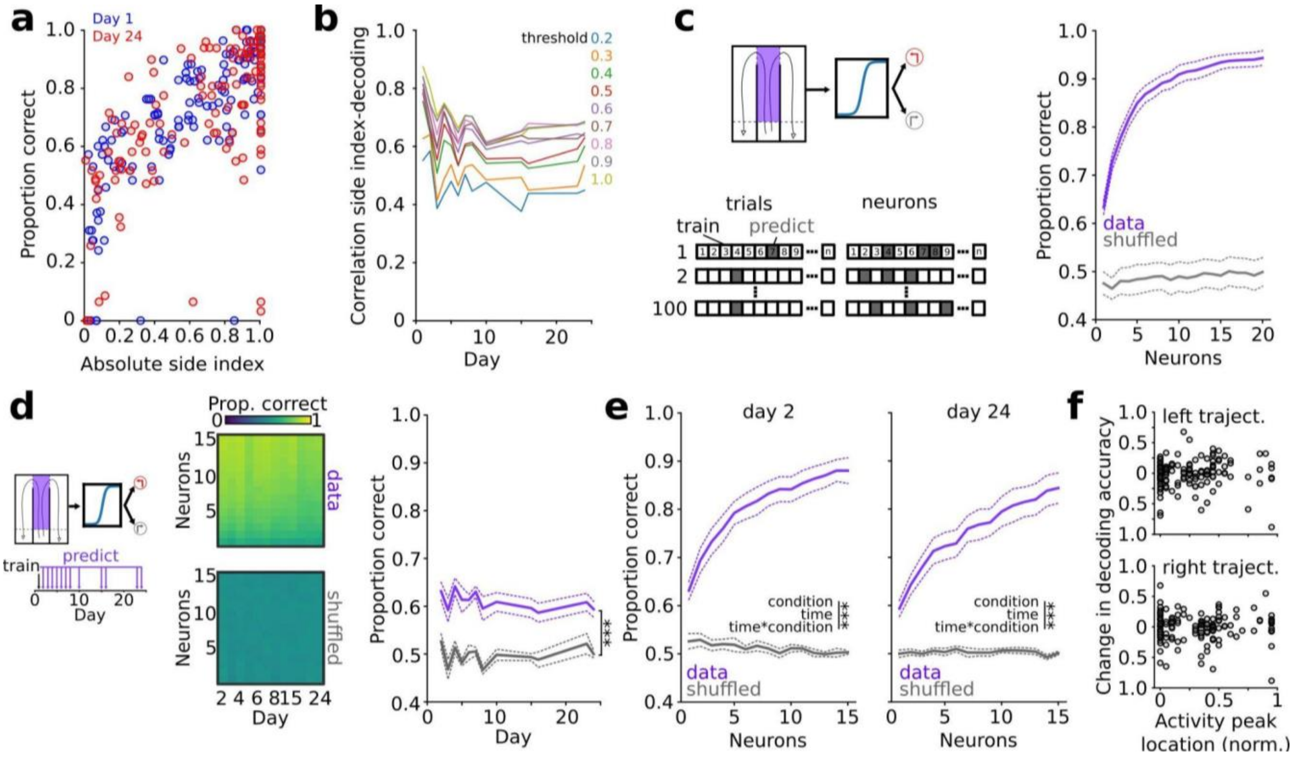

Supplementary Fig. 6: Dependence of decoding accuracy on side index and number of neurons.

a) Decoding performance of the trial outcome prediction using single neurons within single days. Decoding performance correlated with the side index (Day 1 (blue): Spearman's r=0.78, p=10^{-29}, day 24 (red): r=0.63, p=10^{-15}, n=133 neurons with mean activity above 0.5 z).

b) Side index is correlated with decoding performance across days and for different activity thresholds (Spearman's r calculated within each day).

c) Decoding with randomly drawn neurons on day 1. Left: Schematic of the leave-one-out strategy. Right: Decoding accuracy (purple) as a function of neurons used in the model. Performance was significantly better than chance (gray, t=8.67 to 16.22, p=$5*10^{-5}$ to $8*10^{-7}$, 2-way repeated measures ANOVA followed by paired t-tests with Šidák correction) and increased with larger neuron numbers (decoding accuracy-neuron number interaction: F=100.07, p=$3*10^{-15}$, 2-way repeated measures ANOVA).

d) Decoding as a function of randomly selected neurons of the repeatedly active ensemble on subsequent days with models trained on day 1. N=7 mice with >15 neurons in the repeatedly active ensemble. Left: Schematic of decoding strategy. Middle: Colorplots show the decoding performance averaged across mice. Right: Mouse averages of decoding performance based on a single randomly chosen neuron remains significantly different from chance level (F=39.21, p=$8*10^{-4}$). Effect of time: F=2.01, p=0.041; decoding performance*time interaction: F=1.61, p=0.118, 2-way repeated measures ANOVA.

e) Increasing decoding performance with using successively more neurons persists across days. Example plots show decoding performance on day 2 and day 24. In both cases, 2-way repeated measures ANOVA revealed significant effects of decoding performance versus shuffled control data (F=169.93, p=10^{-5} and F=90.63, p=10^{-}

^{5} for day 2 and day 24, respectively) as well as an effect of neuron number (F=25.15, p=10^{-24}; F=35.97, p=10^{-29}) and neuron number*condition interaction (F=56.66, p=10^{-36}; F=25.80, p=10^{-24}). n=7 mice.

f) The change in decoding accuracy of single cells from day 1 to day 24 did not correlate with the position of their peak activity during left (top, Spearman's r=0.134) or right trajectories (bottom, Spearman's r=0.009). n=133 repeatedly active neurons.

Continuous and dotted lines indicate mean ± sem. Source data are provided as a Source Data file.

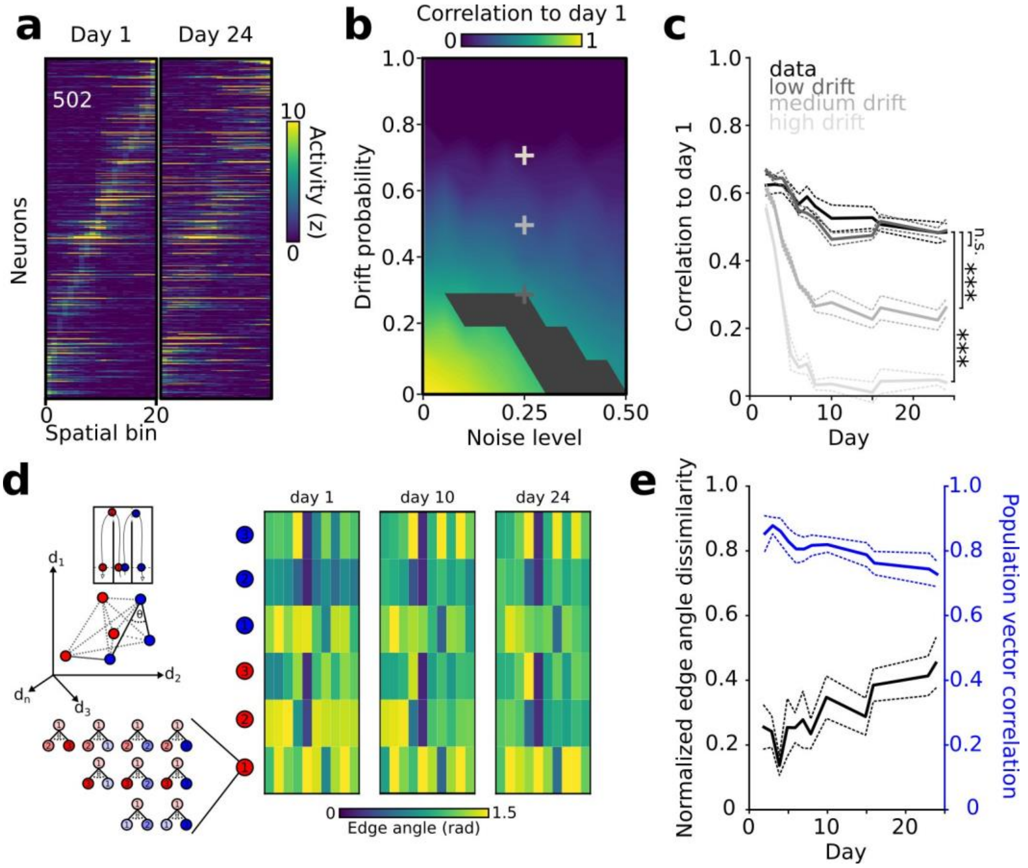

Supplementary Fig. 7: Slow representational drift in the mPFC.

a) Average activity as a function of space during an individual left run of all neurons of the repeatedly active ensemble on day 1 (left) and day 24 (right, n=502). The neurons are shown in the same sorting.

b) Simulated representational drift obtained by cumulatively shifting neuronal tuning functions of day 1 as recording days progress. The resulting correlation of the last recording day to day 1 is shown color-coded as a function of drift probability (i.e., the probability that any given neuron's tuning function drifted by 1 spatial bin between sessions, average of n=8 animals) and the level of noise drawn from a Gaussian distribution added to each neuron's tuning function. The gray area indicates the parameter space in which the correlations of the simulated data is similar to the observed data (±20% of the correlation averaged across mice). Colored symbols show the location in parameter space of the low (30%), medium (50%) and high (70%) drift levels used for analysis over time in c).

c) Comparison of mouse averages of spatial correlation to day 1 (black, same data as shown in Fig. 2d) to the three simulated drifts (gray) indicated in b). True correlations significantly differed for high ($F=259.58$, $p=10^{-7}$) and medium ($F=58.53$, $p=10^{-4}$) but not for low drift ($F=0.14$, $p=0.724$, 2-way repeated measures ANOVAs).

d) Analysis of the geometry of the population response. Left: Population activity was quantified as the mean z-scored calcium signal of all *n* neurons during three of the 20 spatial bins: At the start of the outward trajectory, the midpoint, and the endpoint, separately for left (red) and right (blue) trajectories during repeated task execution. We constructed the geometrical object with corner points given in *n* dimensions by the population

vector, and extracted the edge angles between points (illustrated is one example angle originating from one of the blue corner points). Middle: Example edge angle matrices of the first, 10th and 24th day of one mouse.

e) Edge angle dissimilarity to day 1 increased as a function of days (black, F=2.26, p=0.019). The metric is normalized to vary between 0 (within-day variability estimated from edge angle differences between odd and even runs) and 1 (dissimilarity between shuffled matrices of the first and last day). Similarly, the correlation of population vectors to day 1 (obtained from the same spatial points as edge angles) decayed over time (blue, F=5.53, $p=2*10^{-6}$, one-way repeated measures ANOVAs). Population vector correlation (blue) remained high to the last tested day, indicating the representational drift occurs only slowly in the mPFC network.

Continuous and dotted lines indicate mean ± sem. n=8 mice for all comparisons. Source data are provided as a Source Data file.

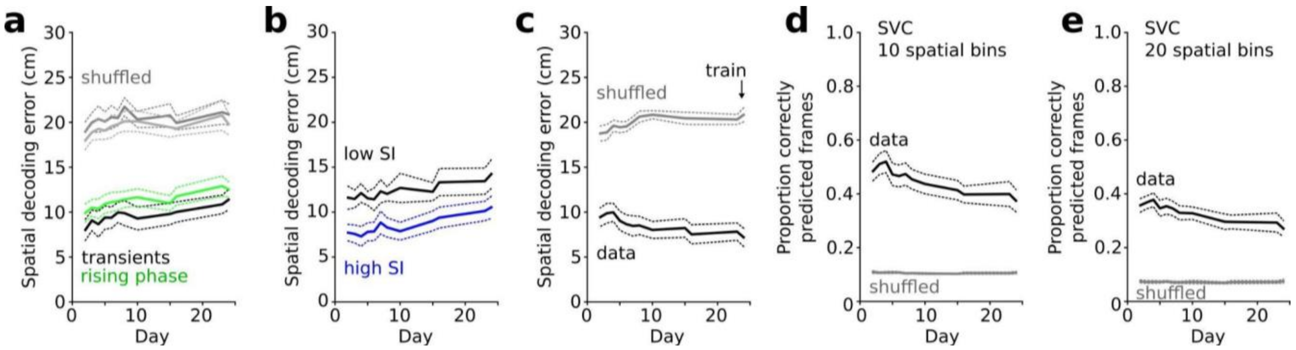

Supplementary Fig. 8: Additional analyses position decoding over days.

a) Decoding position with support vector regression models trained on day 1 using calcium transients (black) or rising phases (green). Transients: main effect of decoding error: F=39.44, p=4*10^{-4}, decoding error-time interaction: F=1.87, p=0.057; rising phase: main effect of decoding error: F=24.38, p=0.002, decoding error-time interaction: F=1.36, p=0.208, 2-way repeated measures ANOVAs. Shuffled data in dark gray are for transients, in light gray for rising phases.

b) Neurons with large SI on day 1 are more effective at position decoding over time (F=28.01, p=0.001) with no decoding error-time interaction effect (F=0.49, p=0.904, 2-way repeated measures ANOVA). The data set was split in 50 % of neurons with largest (blue) and 50 % of neurons with lowest SI (gray). The models were trained on day 1.

c) Linearized position can be decoded over previous days with a model trained on day 24 (black). Decoding performance declined over time (time*condition interaction effect: F=6.14, p=4*10^{-7}) but remained significantly different from shuffled controls (gray, t=-5.69 to -9.46, p=7*10^{-4} to 3*10^{-5}, 2-way repeated measures ANOVA followed by paired t-tests with Šidák correction).

d) Position decoding with an alternative approach using support vector classification (SVC). The model was trained on all frames of day 1. Linearized position was binned in 10 spatial bins. For each imaging frame on subsequent days, the trained model predicted the position bin of the frame. Accuracy was measured as the proportion of correctly predicted frames. While accuracy decreased over days (effect of time: F=5.19, p=5*10^{-6}, time*condition interaction effect: F=5.12, p=6*10^{-6}), it remained significantly above chance level (t=6.24-9.77, p=0.0004-2.5*10^{-5}, 2-way repeated measures ANOVA followed by paired t-tests with Šidák correction).

e) Same as d) but using 20 spatial bins. Effect of time: F=3.96, p=10^{-4}, time*condition interaction effect: F=4.52, p=3*10^{-5}, data vs. shuffled: t=6.15-10.60, p=0.0005-2*10^{-5}, 2-way repeated measures ANOVA followed by paired t-tests with Šidák correction.

For all plots n=8 mice. Continuous and dotted lines indicate mean ± sem. Source data are provided as a Source Data file.

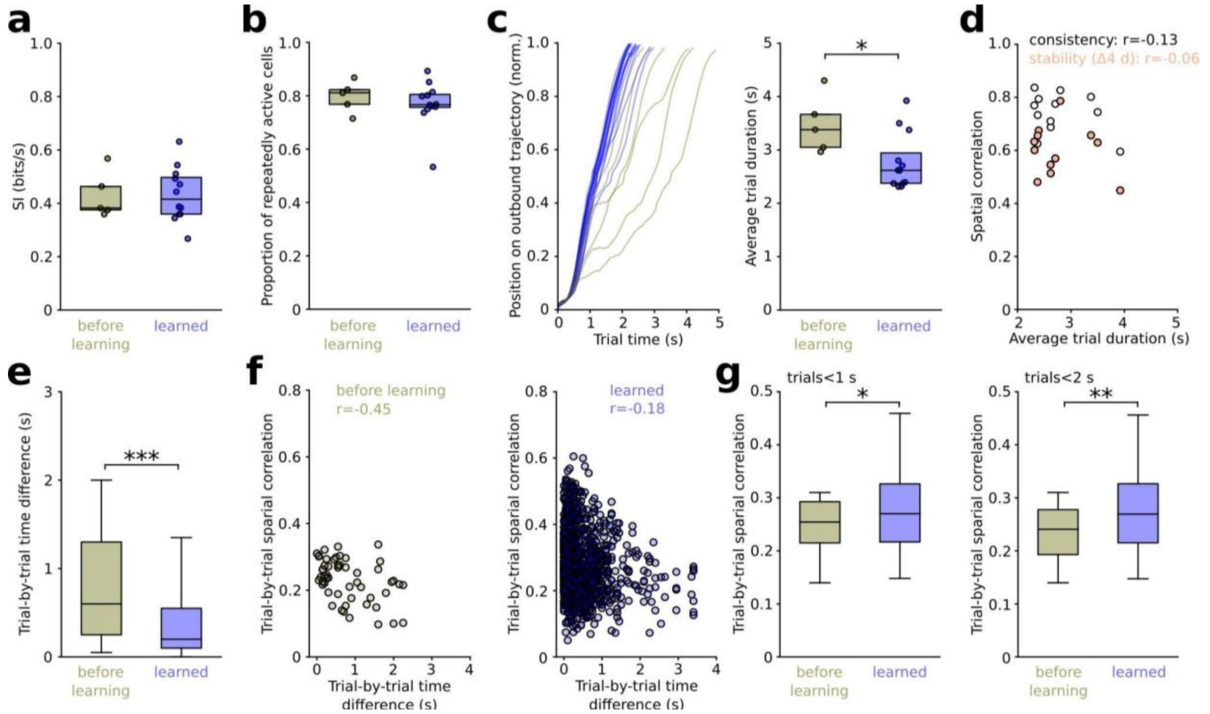

Supplementary Fig. 9: Additional analyses of spatial tuning and behavioral trajectories before learning.

a) Similar average spatial information in before learning (gold) and learned groups (blue) tested on the first day. T=-0.06, p=0.952, n=5 and 12 mice, 2-sided unpaired t-test.

b) Comparable proportion of neurons active during both days that were analyzed to obtain spatial stability. U=38, p=0.442, n=5 and 12 mice, 2-sided Mann-Whitney U-test.

c) Smooth trajectories during outward travel in before learning and learned groups. The example on the left shows individual leftward trajectories from the sampling to the reward zone before and after learning. Right: Learning results in shorter trial durations. U=51, p=0.027, n=5 and 12 mice, 2-sided Mann-Whitney U-test.

d) Average trial duration does neither correlate with spatial consistency (i.e., within day spatial correlation between odd and even runs, p=0.681) nor with spatial stability (measured over t=4 days, p=0.863, Spearman's correlation coefficients). Measurements are from the learned group of mice (n=12). 2-sided correlation tests.

e) Larger behavioral variability in the before learning group. Boxplots summarize the difference in trial-by-trial duration in both groups. T=5.51, p=$4*10^{-8}$, n=53 and 1474 trials, 2-sided unpaired t-test.

f) Trial-by-trial spatial correlation of tuning functions, averaged for all neurons of each trial in the before learning (left) and learned (right) groups, correlates inversely with the difference in duration between the two trials. R-values indicate Spearman's correlation coefficient.

g) Larger trial-by-trial correlations persist when comparing only trials with a maximal difference in duration <1 s (left, t=-2.41, p=0.016, n=36 and 1326 trials) or <2 s (right, t=-3.25, p=0.001, n=48 and 1440 trials, 2-sided unpaired t-tests).

Boxes show median and upper/lower quartiles, whiskers show 5/95 percentiles of the data distribution. Circles within boxes are individual mice. Source data are provided as a Source Data file.

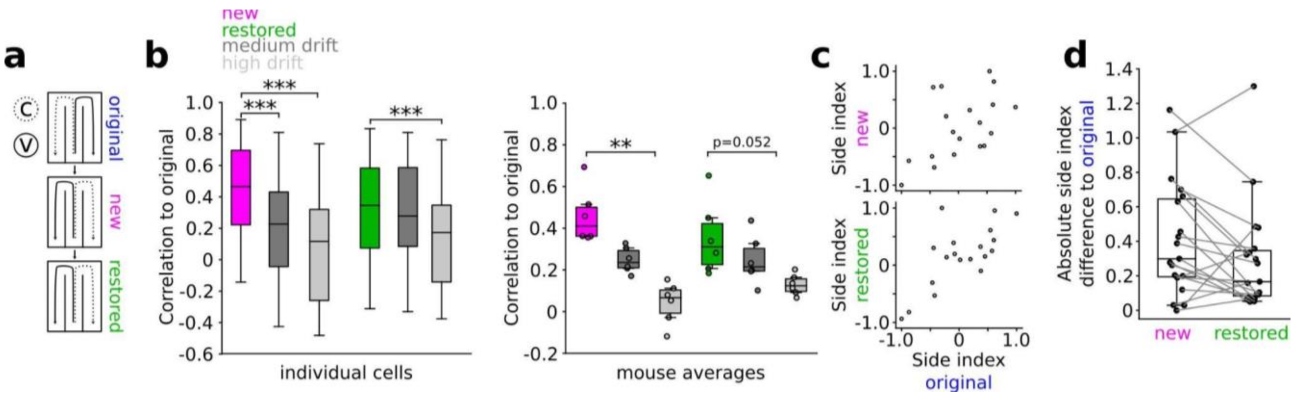

Supplementary Fig. 10: Spatial correlation and trajectory specificity across task contexts.

a) Schematic of original, new and restored rule conditions.

b) Spatial correlation of new (purple) and restored rule conditions (green) to the original rule and to two simulated drift conditions (gray, medium drift=50% probability and high drift=70% probability, see also Supplementary Fig. 7). At the level of individual cells, correlations during new (t=11.07, p=10^{-24}) and restored (t=5.87, p=10^{-8}) remained significant compared to high drift, and correlations during new remained significant compared to medium drift (t=7.09, p=10^{-11}, 2-way repeated measures ANOVAs followed by paired t-tests with Šidák correction, n=272 neurons). On the level of mouse averages, correlations during new remained significant (t=5.51, p=0.003) with a trend during restored compared to high drift. Compared to medium drift: F=3.04, p=0.141, 2-way repeated measures ANOVAs followed by paired t-tests with Šidák correction, n=6 mice).

c) Top: Side index of neurons remains correlated between the original and new rule (Spearman's r=0.542, p=0.016). Bottom: Same as top but for the correlation of side index between original and restored (Spearman's r=0.621, p=0.005). The data are obtained from neurons that were active throughout all three states at >0.5 z mean activity (n=19 neurons).

d) Absolute difference in side index to original does not differ for new and restored conditions. T=48, p=0.060, 2-sided Wilcoxon signed-rank test (n=19 neurons).

Boxes show median and upper/lower quartiles, whiskers show 5/95 percentiles of the data distribution. Circles within boxes are individual mice (b) or cells (c & d). Source data are provided as a Source Data file.

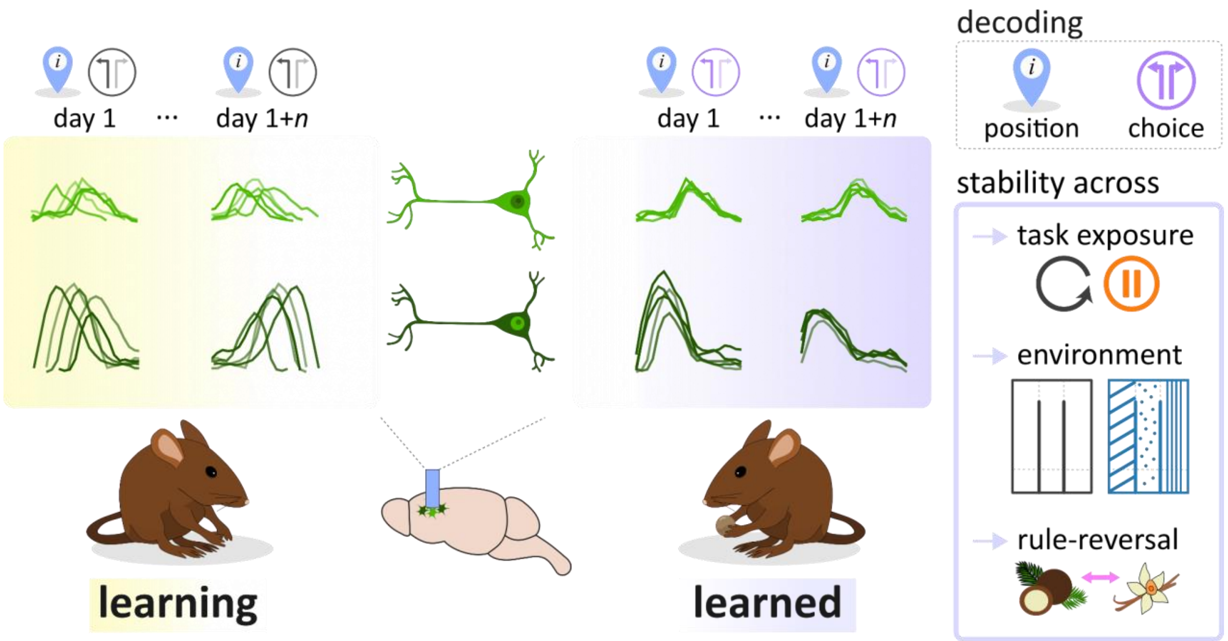

Supplementary Fig. 11: Summary of the main findings of this study.

1-photon miniscope recording of deep layer pyramidal cells in the mouse mPFC during an olfaction guided spatial memory task shows trajectory-specific tuning across days. This tuning is more variable during learning, within a session and across days (left side, highlighted in gold). Nevertheless, position on this trajectory can be decoded, which is however less accurate than in the learned condition. Once the animals have learned the task, tuning is similar across trials and sessions (right side, highlighted in blue). This allows reliable decoding of the animal's position on the trajectory as well as the future choice in the center arm of the arena across days. The stability slightly decreases with time, however pauses in task exposure, modifications of the environment or rule-reversals have little effect on the stability. This summary figure was created with support from Dr. Aurore Cazala.
