# Supplementary material for: A persistent prefrontal reference frame across time and task rules
Source: Nat Commun. 2024 Mar 8;15:2115. doi: 10.1038/s41467-024-46350-4 (PMC10923947; doi:10.1038/s41467-024-46350-4)
Supplement: Supplementary file 3 — Description of Additional Supplementary Files [file 41467_2024_46350_MOESM3_ESM.pdf]

Description of Additional Supplementary Files

File name: Supplementary Movie 1

Description: Olfaction-guided spatial memory task and imaging over weeks. Same mouse in task on day 1 (top) and day 24 (bottom). The left panel shows behavior, the middle panel shows the motion corrected imaging video and the right panel the extracted neuronal calcium signal.
